# Mapping the genomic landscape of multidrug resistance in *Plasmodium falciparum* and its impact on parasite fitness

**DOI:** 10.1126/sciadv.adi2364

**Published:** 2023-11-08

**Authors:** Sachel Mok, Tomas Yeo, Davin Hong, Melanie J. Shears, Leila S. Ross, Kurt E. Ward, Satish K. Dhingra, Mariko Kanai, Jessica L. Bridgford, Abhai K. Tripathi, Godfree Mlambo, Anna Y. Burkhard, Megan R. Ansbro, Kate J. Fairhurst, Eva Gil-Iturbe, Heekuk Park, Felix D. Rozenberg, Jonathan Kim, Filippo Mancia, Rick M. Fairhurst, Matthias Quick, Anne-Catrin Uhlemann, Photini Sinnis, David A. Fidock

**Affiliations:** ^1^ Department of Microbiology and Immunology, Columbia University Irving Medical Center, New York, NY, USA.; ^2^ Center for Malaria Therapeutics and Antimicrobial Resistance, Columbia University Irving Medical Center, New York, NY, USA.; ^3^ Division of Infectious Diseases, Department of Medicine, Columbia University Irving Medical Center, New York, NY, USA.; ^4^ School of Biological Sciences, Nanyang Technological University, Singapore, Singapore.; ^5^ Department of Molecular Microbiology and Immunology, Johns Hopkins Bloomberg School of Public Health, Baltimore, MD, USA.; ^6^ Laboratory of Malaria and Vector Research, National Institute of Allergy and Infectious Diseases, National Institutes of Health, Rockville, MD, USA.; ^7^ Department of Psychiatry, Columbia University Irving Medical Center, New York, NY, USA.; ^8^ Department of Physiology and Cellular Biophysics, Columbia University Irving Medical Center, New York, NY, USA.; ^9^ Division of Molecular Therapeutics, New York State Psychiatric Institute, New York, NY, USA.

## Abstract

Drug-resistant *Plasmodium falciparum* parasites have swept across Southeast Asia and now threaten Africa. By implementing a *P. falciparum* genetic cross using humanized mice, we report the identification of key determinants of resistance to artemisinin (ART) and piperaquine (PPQ) in the dominant Asian KEL1/PLA1 lineage. We mapped *k13* as the central mediator of ART resistance in vitro and identified secondary markers. Applying bulk segregant analysis, quantitative trait loci mapping using 34 recombinant haplotypes, and gene editing, our data reveal an epistatic interaction between mutant PfCRT and multicopy plasmepsins 2/3 in mediating high-grade PPQ resistance. Susceptibility and parasite fitness assays implicate PPQ as a driver of selection for KEL1/PLA1 parasites. Mutant PfCRT enhanced susceptibility to lumefantrine, the first-line partner drug in Africa, highlighting a potential benefit of opposing selective pressures with this drug and PPQ. We also identified that the ABCI3 transporter can operate in concert with PfCRT and plasmepsins 2/3 in mediating multigenic resistance to antimalarial agents.

## Teaser

Genetic mapping identifies the basis of multidrug resistance in a major Southeast Asian lineage of *Plasmodium falciparum* malaria.

## INTRODUCTION

Malaria caused an estimated 247 million cases and 619,000 deaths in 2021, mostly in sub-Saharan Africa (*1*). Efforts to reduce the impact of malaria have repeatedly been stymied by the emergence and spread of *Plasmodium falciparum* resistance to antimalarial drugs (*2*, *3*). Resistance to artemisinin (ART) derivatives, the fast-acting backbone of ART-based combination therapies (ACTs), was first reported in western Cambodia in the late 2000s and has since spread across the Greater Mekong subregion (GMS) (*4*, *5*). In patients, resistance manifests as delayed parasite clearance following ART treatment (*6*). Resistance to piperaquine (PPQ), the long-lasting partner drug of the widely used first-line combination treatment dihydroartemisinin (DHA) + PPQ, was also first detected in western Cambodia and resulted in rapid loss of treatment efficacy across the GMS, with failure rates as high as 87% in northern Thailand (*7*, *8*).

Single-point mutations in Kelch13 (K13) have been identified as the primary mediator of ART resistance in asexual blood stage (ABS) *P. falciparum* parasites (*9*–*12*). Mechanistically, K13 mutations are thought to reduce endocytosis of host hemoglobin, effectively lowering ART activation by hemoglobin-derived heme (*13*–*15*), thus attenuating drug-mediated redox perturbations, protein alkylation, and proteotoxic stress (*16*–*18*). Worryingly, mutant K13 variants have now emerged independently in Rwanda and Uganda, with evidence of delayed parasite clearance as well as increased survival of DHA-treated ring-stage parasites (*19*–*21*). Gene editing studies have identified a substantial role for the parasite genetic background, suggesting contributions from additional determinants (*12*, *22*).

**Fig. 1.**
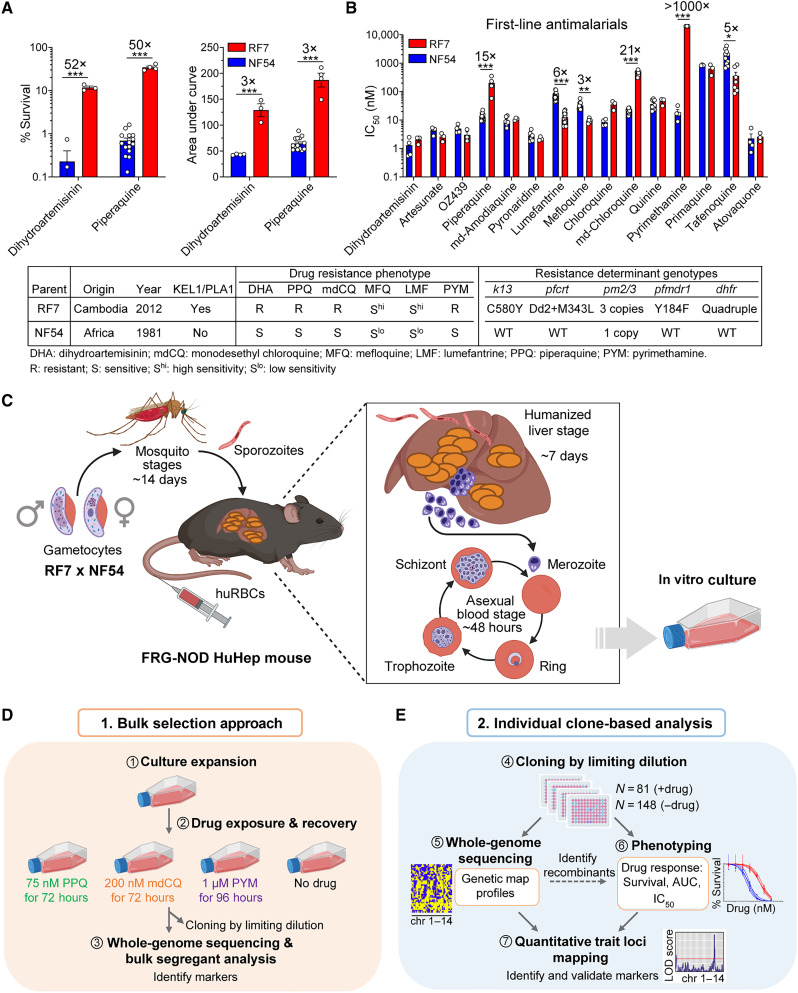
Antimalarial susceptibilities of the genetic cross parents RF7 × NF54 and the experimental workflow for bulk selection versus individual clone-based linkage mapping. (**A**) Bar plots of % survival derived from the DHA ring-stage survival assay (RSA) or the PPQ survival assay (PSA) and the area under the curve (AUC) values, as determined for the genetic cross parents RF7 and NF54. In the RSAs and PSAs, early ring-stage parasites were exposed to the pharmacologically relevant concentrations of 700 nM DHA for 4 hours or 200 nM PPQ for 72 hours, respectively, and survival was measured as a percentage of mock-treated cultures. (**B**) IC_50_ values of RF7 and NF54 parasites exposed for 72 hours to first-line antimalarials. Values represent the means ± SE (*N* = 3 to 17, *n* = 2). Statistical significance was determined by unpaired Student’s *t* tests, with a Holm-Sidak post hoc test to correct for multiple comparisons. The IC_50_ fold shifts are indicated above the bars. *P < 0.05, **P < 0.01, ***P < 0.001. Numbers listed above the statistics indicate the fold differences between parental IC_50_ values. The table summarizes parental phenotypic and genotypic characteristics. WT, wild-type. Mutant genotypes include the following: Dd2 *pfcrt* mutations: M74I, N75E, K76T, A220S, Q271E, N326S, I356T, and R371I; quadruple *dhfr* mutations: N51I, C59R, S108N, and I164L. (**C**) Overview of the genetic cross pipeline performed in humanized FRG-NOD mice. huRBCs, human red blood cells. (**D**) Bulk selection approach used for bulk segregant analysis. (**E**) Individual clone-based linkage approach used to identify genetic determinants of drug resistance. Images were created with BioRender.com.

Investigations into PPQ resistance initially identified multicopy *plasmepsins 2* and *3* (*pm2/3*) as biomarkers (*23*, *24*). These aspartic proteases contribute to hemoglobin proteolysis inside the parasite’s acidic digestive vacuole (DV), thereby releasing globin (a source of amino acids) and toxic heme. Subsequent studies of PPQ resistance identified a critical role for mutations (such as M343L or F145I) in the DV membrane-resident chloroquine resistance transporter (PfCRT). These single-point mutations emerged in the GMS on the background of the Southeast Asian mutant PfCRT Dd2 isoform [Fig. 1, legend; (*25*–*28*)], which is known to mediate resistance to CQ, a 4-aminoquinoline that is chemically related to the bis-quinoline PPQ. This isoform also partially reduces parasite susceptibility to amodiaquine while increasing susceptibility to lumefantrine (LMF), both widely used ACT partner drugs (*18*, *29*–*31*). PfCRT variants (including M343L, F145I, or other mutations) are thought to mediate PPQ resistance by enabling drug efflux through the central cavity of PfCRT (*32*, *33*). This efflux is thought to remove PPQ from the DV, where it normally accumulates as a protonated species and inhibits heme detoxification and hemozoin formation (*34*–*36*). PPQ-resistant parasites display an unusual dose-response profile in which the IC_50_ (i.e., the concentration that yields half-maximal growth inhibition) shows minimal differences relative to PPQ-sensitive parasites, yet at high PPQ concentrations parasites show a biphasic survival curve (*37*). Defining the relative contributions of PfCRT and *pm2/3*, in terms of resistance and fitness costs, has until now been elusive.

*P. falciparum* genetic crosses using simian models have previously proven effective in identifying drug resistance determinants, including *pfcrt* and antifolate resistance mediators (*38*). Recent studies have explored ART or CQ resistance using the FRG-NOD humanized mouse model that permits pre-erythrocytic liver stage development and infection of human red blood cells (RBCs) by *P. falciparum* parasites (*39*–*43*). To further investigate the genetic basis of *P. falciparum* resistance to DHA and PPQ, we conducted a genetic cross between a Cambodian multidrug-resistant parasite and a drug-sensitive African parasite. Our data uncover secondary markers of ART resistance and elucidate the epistatic relationship between *pfcrt* and *pm2/3* in modulating PPQ resistance. We also identify the ABC transporter (*abci3*) as an additional member of this gene interaction network involved in differential susceptibility to other antimalarial compounds. Finally, we identify PPQ-resistant *pfcrt* as a driver of enhanced sensitivity to LMF and mefloquine (MFQ), supporting their combined use in the field.

## RESULTS

### Implementation of a RF7 × NF54 genetic cross to map determinants of drug resistance

To identify determinants of *P. falciparum* resistance to DHA and PPQ, we conducted a genetic cross between the PPQ- and DHA-resistant contemporary Cambodian clinical isolate RF7 and the sensitive African parasite line NF54 (Fig. 1). RF7 was selected as representative of the multidrug-resistant KEL1/PLA1/PfPailin lineage that has dominated the GMS (*5*, *10*). RF7 expresses the K13 C580Y variant as well as the PfCRT Dd2 + M343L isoform and multicopy *pm2/3*, with a prior study confirming *pm2/3* overexpression (*27*). We first characterized RF7 and NF54 susceptibility profiles to DHA, PPQ, and a panel of other antimalarials (Fig. 1A). Using the ring-stage survival assay (RSA) to measure ART resistance in vitro, we observed a high percentage of surviving DHA-treated parasites (mean ± SE, 11.8 ± 1.0%), whereas NF54 showed marginal survival (0.2 ± 0.2%). Likewise, using the PPQ survival assay (PSA), ring-stage RF7 parasites gave a high survival rate (34.5 ± 2.6%), contrasting with minimal survival (0.7 ± 0.1%) in NF54 (Fig. 1A and table S1). RF7 parasites showed elevated Area Under the Curve (AUC) values for both DHA and PPQ (Fig. 1A).

RF7 was also highly resistant to monodesethyl chloroquine (mdCQ; the active metabolite of CQ) and pyrimethamine (PYM). In contrast, RF7 exhibited three- to sixfold increased sensitivity to the ACT partner drugs, MFQ and LMF, compared to NF54 (Fig. 1B and table S1). There were no significant differences in IC_50_ values for other antimalarials tested, including amodiaquine, pyronaridine, quinine and atovaquone.

To achieve a genetic cross, RF7 and NF54 gametocytes were fed as 1:1 pools to female *Anopheles stephensi* mosquitoes to initiate sexual stage recombination. We then infected four human liver-chimeric FRG-NOD mice with sporozoites, either via intravenous inoculation of manually dissected sporozoites or via mosquito bites (Fig. 1C and table S2). Haploid ABS parasites were recovered on day 7.5 postinfection and subsequently cultured in vitro (Fig. 1C).

### Bulk segregant analyses identify major determinants of *P. falciparum* resistance to PPQ, CQ, and PYM

Whole-genome sequencing (WGS) of the RF7 and NF54 parents identified 18,489 (“18k”) single-nucleotide polymorphisms (SNPs) between their core genomes (tables S3 and S4). These SNPs were located in 2439 *P. falciparum* coding genes, corresponding to nearly half of the core genome of ~5100 genes, and with an average of ~1 kb distance between SNPs. These SNPs were distributed quite evenly across coding and noncoding regions (fig. S1, A to C). The extensive diversity between these geographically distinct parents enabled high-resolution genetic mapping of drug susceptibility profiles in the cross progeny.

**Fig. 2.**
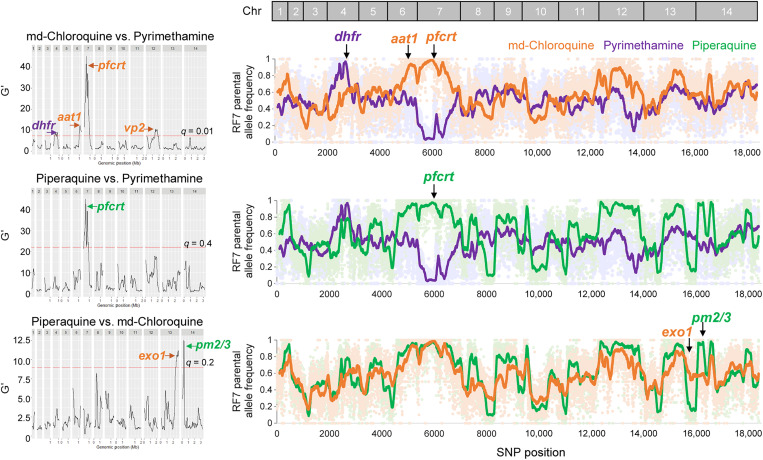
Bulk segregant analyses of progeny pools pressured with PPQ, mdCQ, or PYM. The significant genetic loci enriched by each drug in individual drug-drug pairwise comparisons are shown with black lines representing G′ values (left), along with their corresponding RF7 parental allele frequencies for the set of 18k SNPs that differ between the two parents (right). The G′ is the smoothed statistical value calculated for individual SNPs and used for detecting QTLs. Regions with a false discovery rate *q* value < 0.01 (mdCQ versus PYM), < 0.4 (PPQ versus PYM), or < 0.2 (PPQ versus mdCQ) were considered statistically significant QTLs (table S5). Genes enriched by each drug and the RF7 parental allele frequency for the drug-treated samples are colored as follows: mdCQ (orange), PYM (purple), and PPQ (green). Individual allele frequencies for each drug-treated pool are indicated by light dots in the background. The solid colored lines represent the averaged RF7 allele frequency across windows of 100 consecutive SNPs.

We applied a bulk selection approach to enrich for recombinant ABS progeny resistant to PPQ, mdCQ, or PYM (Fig. 1D). Surviving parasites from these drug-pressured bulk cultures were then subjected to WGS,and parental allele frequencies were measured across the 14 chromosomes (Fig. 1D). Using bulk segregant analysis, we then compared the parental frequencies at each of the 18k SNP positions between drug-treated cultures or between drug-treated and untreated cultures (Fig. 2 and fig. S2). Comparing the PYM-treated bulk against the mdCQ-treated sample or the untreated bulk revealed a significant quantitative trait locus (QTL) on chromosome (chr) 4 (Fig. 2, table S5, and fig. S2A). This locus, spanning 141 kb, contained dihydrofolate reductase (*dhfr*), which in RF7 parasites encodes a quadruple mutant PYM-resistant allele. By comparison, mdCQ selected for a dominant 856-kb QTL on chr7 that contains the mutant *pfcrt* Dd2 + M343L allele, as well as two smaller peaks on chr6 and chr12 that contain the putative amino acid transporter *aat1* and the v-type pyrophosphatase *vp2*. mdCQ and PPQ showed similar allelic frequency profiles, consistent with their similar modes of action (*35*). PPQ, however, selected for a 117-kb chr14 segment containing the tandem *pm2/3* amplicon. In contrast, PPQ selected against a 484-kb segment on chr13 harboring mutant E415G exonuclease-I, which had previously been proposed to be a molecular marker of PPQ resistance (Fig. 2 and fig. S2) (*24*).

Although bulk segregant analysis can identify loci containing the major resistance mediators for these drugs, this approach may be constrained by the fitness cost of certain genotypes that associate with reduced expansion in bulk cultures. To refine the identification of resistance markers and map secondary determinants, as well as understand epistatic interactions between genes, we performed QTL analysis with individual clones (Fig. 1E).

### Drug pulses enrich for genetically and phenotypically diverse progeny

#### WGS analysis identifies a set of genetically distinct recombinant progeny

**Fig. 3.**
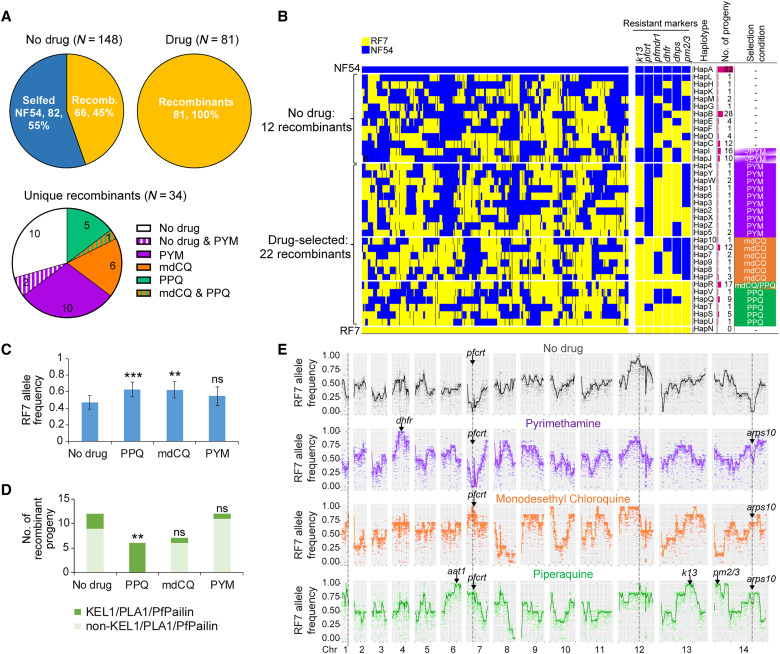
Genetic analysis of recombinant progeny and enrichment of diverse progeny using drug pulses. (**A**) Number and percentage of selfed progeny versus genetic recombinants obtained from cloning in the absence of drug or following drug exposure. Distribution of the 34 unique recombinants obtained with no drug and/or selected by PPQ, mdCQ, or PYM. (**B**) Allelic map for the 34 recombinant progeny and two parents, grouped by drug treatment condition. Shown are the parental alleles inherited by each progeny for 14,476 SNPs (after excluding SNPs missing in any of the 34 recombinant haplotypes), the genotypes of known resistance markers, and the number of derived progeny clones per haplotype sorted by selection condition. (**C**) Mean RF7 allele frequency inherited by the 34 recombinant progeny clones for each drug treatment group. (**D**) Count of KEL1/PLA1/PfPailin haplotypes in progeny clones that were selected by each drug condition. Statistical significance was tested for the drug-selected progeny clones against the “no drug” group, using Fisher’s exact test. **P < 0.01; ***P < 0.001; ns, not significant. (**E**) Frequency of RF7 parental allele in progeny clones derived posttreatment with PPQ (*N* = 6), mdCQ (*N* = 7), or PYM (*N* = 12) or in the absence of drug (*N* = 12). Individual allele frequencies (light dots in the background) are reported as a weighted allele frequency across the number of clones for each group. The solid colored lines represent the averaged RF7 allele frequency across windows of 100 consecutive SNPs. The skews in allele frequency at specific loci in the genome are indicated by dashed lines.

We cloned 148 individual progeny by limiting dilution of untreated cultures and identified 66 recombinant progeny using WGS analysis of the 18k SNP set. The remaining 82 progeny proved to be genetically identical to NF54, an indication of a high selfing rate for this parent in our cross. The 66 recombinant clones that were derived in the absence of drug segregated into 12 unique haplotypes. Eight recombinant haplotypes were recovered from the one mouse that was intravenously inoculated with sporozoites, whereas only six recombinant haplotypes were recovered from the three mice infected by mosquito bites (Fig. 3A and table S2). An additional 81 recombinant clones were obtained from bulk cultures pressured with PPQ, mdCQ, or PYM, yielding an additional 22 unique haplotypes (Fig. 3, A and B). Our analysis focused on these 34 genetically distinct haplotype groups (Fig. 3B), in addition to parental NF54 and RF7 (referred to as HapA and HapN, respectively). Unexpectedly, we observed minimal overlap in the sets of recombinant progeny selected using PPQ and mdCQ pressure. Also, no common haplotypes were obtained using PYM pressure compared with either of the other two drugs (Fig. 3B). PPQ and mdCQ generated five and six unique recombinant haplotypes, respectively, and one additional shared haplotype. This finding suggests that resistance to each drug requires distinct combinations of genetic determinants.

#### Genetic analysis reveals recombination rates, inheritance patterns, and relatedness among progeny

Our analysis of the 34 unique recombinants revealed a recombination rate of 15.1 kb/cM and a positive correlation (*R*^2^ = 0.47) between the number of crossover events and physical lengths (table S6 and fig. S1, D and E), similar to previous crosses [12.1 to 14.3 kb/cM; (*40*, *44*, *45*)]. Most clones shared ~50% similarity in parental allelic composition across the genome, as measured by identity-by-descent analysis (fig. S1F). Nonetheless, using multidimensional scaling of pairwise genetic distances, we observed that the recombinant progeny clustered based on their selection conditions (fig. S1G). Progeny derived with PPQ or mdCQ pressure clustered more closely and were distinct from the progeny pressured with PYM or derived without drug. This observation agrees with our bulk segregant results and aligns with our expectations based on drug chemical relatedness. There was also significantly higher inheritance of RF7 SNPs in the recombinants obtained following PPQ or mdCQ exposure compared to those obtained in the absence of any drug pressure (Fig. 3, B and C, and fig. S1H), suggesting the possibility of multiple determinants existing in linkage disequilibrium.

#### PPQ and mdCQ show differential selective pressure for the KEL1/PLA1/PfPailin haplotype

We observed that all progeny selected by PPQ were of the KEL1/PLA1/PfPailin haplotype (*k13* C580Y, *k13*-flanking region, and multicopy *pm2/3*), identical to RF7 (Fig. 3D, fig. S3A, and table S7). However, only one of the seven mdCQ-selected progeny inherited this haplotype. While mdCQ treatment enriched for progeny clones harboring mutant *pfcrt*, this drug selected against a segment on chr14 encompassing *pm2/3* (Fig. 3B). This observation suggests that amplified *pm2/3* may have a fitness cost in the presence of RF7 mutant *pfcrt*. Likewise, the majority of recombinants obtained under PYM or drug-free conditions did not harbor the KEL1/PLA1/PfPailin lineage (Fig. 3D). This haplotype’s significant association with PPQ-selected progeny (*P* = 0.009, Fisher’s exact test), but not with the mdCQ or PYM-selected progeny, suggests that PPQ favors this particular lineage.

#### Genomic segregation distortions map to mutant pfcrt and a chr14 loci

To identify loci under segregation distortion in the progeny, we aggregated the distinct sets of clones recovered without or with drug selection in silico and examined their allele frequencies. In the drug-negative clones, there was extreme inheritance bias toward NF54 wild-type (WT) SNPs at multiple loci on chromosomes 1, 7, 13, and 14. These skews were reversed by PPQ, mdCQ, or PYM treatments, which enriched for RF7 SNPs in these segments (Fig. 3E). This reversal was most prominent for *pfcrt* found on chr7 in mdCQ- or PPQ-pressured parasites, implying a strong fitness cost for mutant *pfcrt* parasites in the absence of selective pressure. Using the “pooled” drug-selected progeny clones, we also identified an additional 251-kb segment on chr14 containing *arps10*, which was enriched by all three drugs (Fig. 3E). In addition to *pfcrt*, the PPQ-selected clones showed 100% inheritance of RF7 SNPs for chr14 (377 kb) and chr13 (516 kb) segments containing *pm2/3* and *k13*, respectively (Fig. 3E). This observation supports our finding that PPQ positively selected for the KEL1/PLA1/PfPailin lineage. In addition, PPQ, but not mdCQ or PYM, selected for a 416-kb chr6 segment encompassing *aat1*, suggesting a possible involvement of this marker with PPQ resistance.

#### Drug exposure enriches for progeny with diverse phenotypic responses

**Fig. 4.**
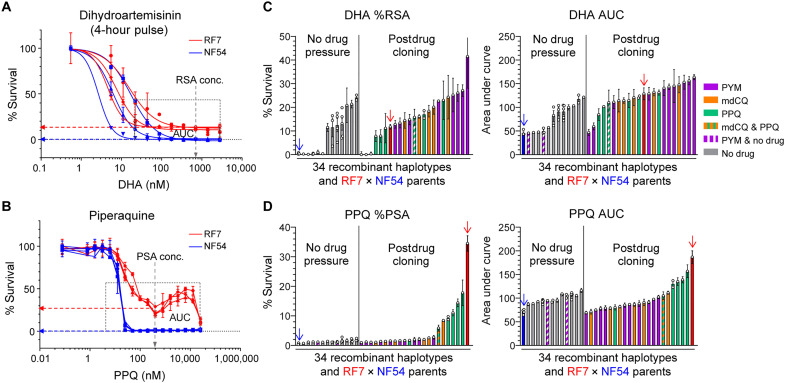
Phenotypic response of parents and progeny to DHA and PPQ. (**A** and **B**) Dose-response curves for RF7 and NF54 parents across a range of DHA and PPQ concentrations (*N =* 3, *n* = 2). The percent survival at the RSA and PSA concentrations of 700 nM DHA and 200 nM PPQ for 4 or 72 hours, respectively, were used to determine DHA and PPQ resistance levels, whereas AUC values were measured as total survival across a range of concentrations (22 nM to 2.8 μM for DHA and 1.6 nM to 25.6 μM for PPQ). (**C** and **D**) DHA and PPQ response measured by %RSA and DHA AUC values (C) and %PSA and PPQ AUC values (D), respectively, in the 34 independent recombinant haplotypes and two parents. Progeny clones were obtained either in the absence of drug pressure (gray) or after selection with PYM (purple), mdCQ (orange), or PPQ (green). Data are plotted alongside RF7 (red arrow) and NF54 (blue arrow). Each bar represents the mean percent survival ± SE or AUC ± SE for a recombinant haplotype profiled in four independent experiments with technical duplicates. Haplotypes are ordered by the parasites’ resistance levels for each drug metric. For certain haplotype groups in which two or more identical clones were obtained, multiple points depict phenotypic results for more than one sibling progeny.

To characterize the DHA and PPQ phenotypic responses of the 34 recombinant haplotypes, we profiled a panel of representative progeny and their RF7 × NF54 parents in drug susceptibility assays (Fig. 4 and table S8). We observed a wider range of phenotypic responses and higher survival and AUC values to either DHA or PPQ for the clones derived post drug selection compared to the nondrug pressured group (Fig. 4, C and D). Notably, several of these drug selected progeny clones had even higher DHA RSA survival and AUC values compared to parental RF7. This result was unexpected as the highly DHA-resistant progeny were derived post-PYM selection. For PPQ, five of the six PPQ-selected clones showed high levels of PPQ survival and resistance, yet none had higher PSA survival than the parental RF7. This finding suggests a large fitness cost even among the PPQ-resistant progeny and reinforces the importance of applying drug pressures to diversify genetic cross progeny phenotypes.

### DHA resistance associates with *k13* and other secondary determinants

#### QTL mapping of DHA resistance reveals k13 as the primary determinant

**Fig. 5.**
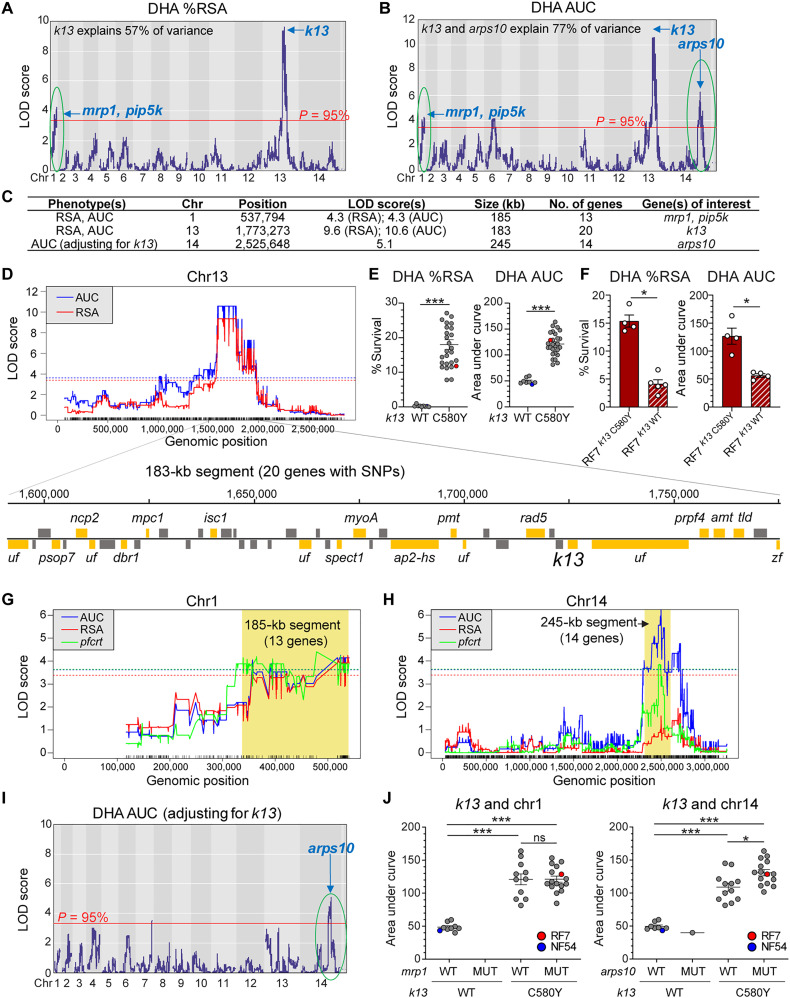
QTL mapping of DHA responses in progeny identifies *k13* as the primary DHA resistance locus. (**A** and **B**) LOD plots for %RSA and AUC levels showing the significant QTLs above the 95% probability threshold (red line). (**C**) List of QTL segments for %RSA and AUC levels. (**D**) Genes in QTL segment on chr13, with those having nonsynonymous mutations between RF7 and NF54 colored in orange (*n* = 20) or gray where mutations were absent in RF7 and NF54. Gene names are as listed. uf, unknown function. (**E**) The %RSA and AUC levels in recombinant progeny segregated by *k13* parental allele, C580Y (in RF7) and WT (in NF54). Significance was tested using Mann-Whitney *U* tests. ***P < 0.001. (**F**) DHA responses in *k13*-edited isogenic RF7 clones showed that the *k13* WT allele reduces %RSA and AUC levels. Bars represent the means ± SE (*N* = 4, *n* = 2). Significance was tested using Mann-Whitney *U* tests. *P < 0.05. (**G** and **H**) Linkage of chr1 (G) and chr14 (H) segments with *pfcrt* from QTL analysis using the *pfcrt* genotype as an outcome. Shown are the significant QTLs above the 95% probability threshold for each analysis. (**I**) LOD plot for AUC levels after adjusting for *k13* as a covariate suggests independent inheritance of the chr14 segment with *k13* and coinheritance of the chr1 segment with *k13*. (**J**) Scatterplot of AUC levels in progeny segregated by *k13*, *mrp1* (chr1), and *arps10* (chr14) genotypes, showing that the RF7 chr14 segment is associated with increased AUC levels. We note that none of the progeny harbored MUT *mrp1* and WT *k13* alleles. Significance between groups was tested using Mann-Whitney *U* tests. *P < 0.05, ***P < 0.001. MUT, mutant.

We next performed linkage analysis to uncover QTLs associated with DHA resistance, using the phenotype-genotype data from the progeny clones (tables S4 and S8). There was a good correlation (*R* = 0.85) between RSA and AUC values (fig. S4). QTL mapping using either sets of values revealed a dominant peak on chr13. This 183-kb segment contained 109 SNPs including 47 nonsynonymous mutations within 20 genes and included *k13*, the primary ART resistance mediator (Fig. 5, A to D). We also observed coinheritance of this segment, spanning −133 to +47 kb of the *k13* gene, in all mutant progeny (fig. S3). This observation suggests the possible presence of additional loci that have an impact on ART resistance or that compensate for altered mutant K13 function. Grouping the recombinant progeny by their *k13* genotypes (C580Y versus WT) showed a clear segregation in DHA response, with mutant progeny showing higher RSA survival and AUC levels (Fig. 5E). Reverting C580Y to WT in RF7 parasites using CRISPR-Cas9–based gene editing resulted in a significant reduction in RSA and AUC levels (from 15.9 to 4.6% and from 126.6 to 56.3, respectively; Fig. 5F). By comparison, NF54 RSA and AUC levels were 0.2% and 43.3, respectively (Fig. 1A and table S8).

#### QTL mapping uncovers potential secondary determinants on chr1 and chr14

In addition to the chr13 segment, we observed two smaller QTLs on chr1 and chr14 that were significantly associated with DHA resistance (Fig. 5, A, B, G, and H, and table S9). We then examined whether these associations were additive or epistatic in modulating DHA response. Applying *k13* as a covariate resulted in the loss of the chr1 peak for both RSA and AUC, suggesting that the chr1 segment was likely coinherited with *k13* (Fig. 5I and fig. S5A). This was evident by the lack of any difference in RSA levels when stratified by the *k13* and chr1 genotypes (Fig. 5J). In contrast, the chr14 segment showed a significant QTL peak for AUC either when correcting for *k13* genotypes or in the subset of *k13* mutants (Fig. 5I, fig. S5B, and table S9). Mutant *k13* progeny exhibited higher mean AUC levels when harboring RF7 alleles for chr14, suggesting that this segment might enhance parasite survival at higher doses of DHA (Fig. 5J and table S9).

Within the chr1 185-kb segment, we observed nonsynonymous mutations in 13 genes, including the drug-resistant candidates *mrp1* and *pip5k* (table S9). The 245-kb segment on chr14, notable in only the AUC outcome, comprised 14 genes including *arps10*. Examination of sequence diversity in *mrp1*, *pip5k*, and *arps10*, across the set of ~2500 sequenced *P. falciparum* genomes (*46*), suggested geographic differences with the RF7 parental mutations being prevalent in clinical isolates from Cambodia, Mali, and Ghana (fig. S5C). These chr1 and chr14 segments also showed linkage disequilibrium with *pfcrt* (Fig. 5, G and H, and fig. S5, D and E).

### PPQ resistance is conferred by mutant Dd2 + M343L *pfcrt* and multicopy *pm2/3*

**Fig. 6.**
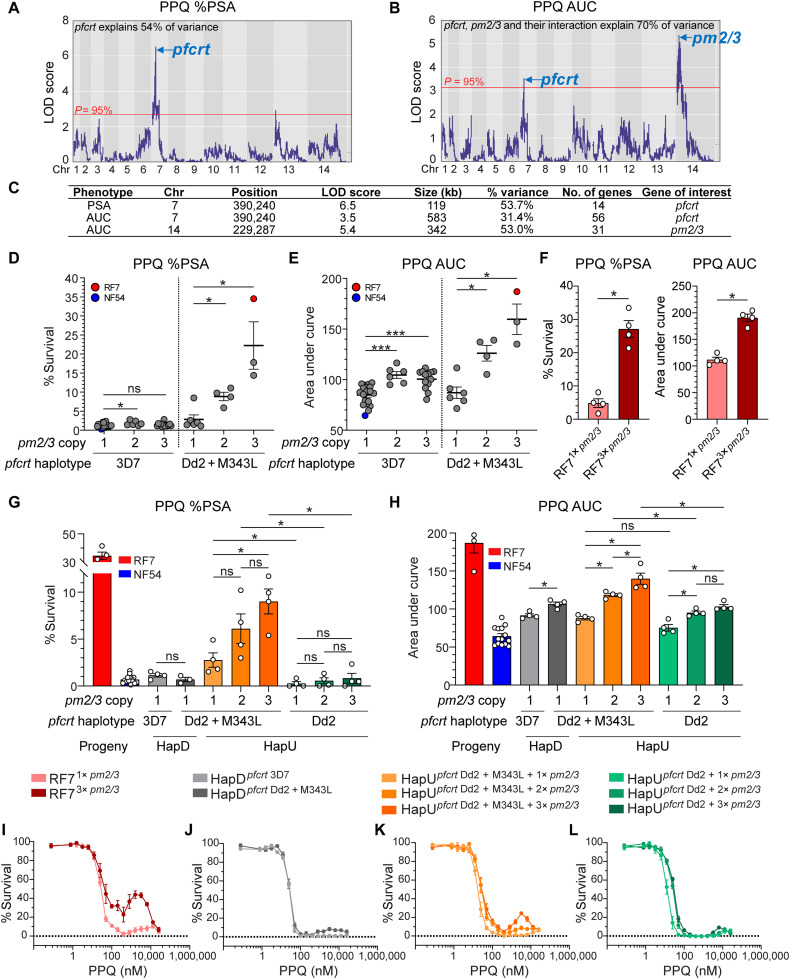
QTL mapping of PPQ resistance in progeny and response in *pfcrt*-edited progeny reveal an association of PPQ resistance with mutant Dd2 + M343L *pfcrt* and multicopy *pm2/3*. (**A** and **B**) LOD plots for %PSA and AUC levels showing QTLs detected above the 95% probability threshold (red line). (**C**) List of QTL segments for %PSA and AUC levels. (**D** and **E**) Scatterplot of the %PSA or AUC levels for 34 independent recombinant progeny and parents, segregated by *pm2/3* copy and *pfcrt* genotypes. NF54, 3D7 *pfcrt*; RF7, Dd2 + M343L *pfcrt*. Significant differences in PPQ response between the recombinant groups harboring different *pm2/3* copies were tested by Mann-Whitney *U* tests. *P < 0.05, ***P < 0.001. (**F**) PPQ response in isogenic RF7 clones containing one versus three copies of *pm2/3* in the mutant Dd2 + M343L *pfcrt* background, showing that single-copy *pm2/3* reduces %PSA and AUC levels. Bars represent the means ± SE (*N* = 4, *n* = 2). Significance was tested using Mann-Whitney *U* tests. *P < 0.05. (**G**) PPQ %PSA and (**H**) AUC levels in *pfcrt*-edited HapD progeny (3D7 versus Dd2 + M343L alleles) with single *pm2/3* copy, and in *pfcrt-*edited HapU progeny (Dd2 versus Dd2 + M343L alleles) with either one, two, or three copies of *pm2/3*. Bars represent the means ± SE (*N* = 4, *n* = 2). Significance between the isogenic edited progeny lines was tested using Mann-Whitney *U* tests. *P < 0.05. The colored key for parasite lines applies to (G) to (L). (**I** to **L**) Dose-response curve in RF7 clones [I; colored the same as (F)], HapD (J), unedited HapU (K), and *pfcrt-*edited HapU (L) progeny, showing that multicopy *pm2/3* and mutant *pfcrt* are necessary for the PPQ biphasic response. Each line depicts the mean percent parasite survival ± SE (*N* = 3 to 4, *n* = 2). In parallel studies, serum was noted to lower %PSA (fig. S8, B and C).

To identify the markers of PPQ resistance, we performed QTL mapping using the recombinant progeny values obtained from the PSA and AUC data (table S8). Both datasets identified a major QTL on chr7, which mapped to *pfcrt* (Fig. 6, A to C, and table S10). Our AUC analyses also identified an additional locus on chr14 harboring the conserved *pm2/3* amplicon (Fig. 6, B and C; fig. S6A; and table S10). Controlling for *pfcrt* as a covariate revealed no other peaks in the PSA analysis, suggesting that the *pfcrt* RF7 allele is the main determinant of survival at 200 nM PPQ (fig. S6B). In contrast, controlling for *pfcrt* improved the logarithm of the odds (LOD) score for the *pm2/3* segment for AUC. Reciprocally, controlling for *pm2/3* revealed a higher LOD score for the *pfcrt* segment (fig. S6B). Our results suggest a link between *pfcrt*, *pm2/3*, and PPQ AUC.

#### The epistatic interaction between mutant pfcrt and multicopy pm2/3 modulates PPQ resistance levels

Examining the PPQ response in WT *pfcrt* progeny revealed a small but significant association between *pm2/3* amplifications and higher AUC levels (Fig. 6, D and E). Mutant *pfcrt* progeny harboring multicopy *pm2/3* showed a larger increase in both PSA and AUC levels, coinciding with a biphasic response at high PPQ concentrations (fig. S7). These findings suggest an epistatic interaction between *pm2/3 and pfcrt* in modulating high-grade PPQ resistance.

To further examine the relative contributions of *pm2/3* and *pfcrt*, we took advantage of a spontaneous loss of *pm2/3* copy number in the RF7 parental line that occurred over ~3 months of continuous in vitro culture and used limiting dilution to obtain clones with three copies or a single copy of *pm2/3*. The multicopy *pm2/3* clone exhibited significantly higher PSA and AUC levels and displayed a biphasic PPQ dose-response curve (Fig. 6, F and I, and fig. S8). These data confirm that *pm2/3* amplifications can augment PPQ resistance in the presence of mutant *pfcrt*.

We validated these markers by editing *pfcrt* in progeny that corresponded to two unique haplotypes and differed in their *pm2/3* copy numbers. The HapD progeny was chosen as it represented a DHA-resistant, PPQ-sensitive line. Introducing the mutant Dd2 + M343L *pfcrt* allele into HapD WT *pfcrt* parasites having a single copy of *pm2/3* did not affect PSA levels and led to a minor increase in AUC levels, suggesting that this mutant *pfcrt* allele did not suffice to confer PPQ resistance in this progeny (Fig. 6, G, H, and J).

We also selected HapU as it had a KEL1/PLA1/PfPailin haplotype, was DHA and PPQ resistant, and exhibited the highest PPQ resistance level amongst our recombinant progeny (tables S7 and S8). As with RF7, this line was observed to spontaneously deamplify *pm2/3* and limiting dilution yielded HapU clones with one to three *pm2/3* copies. We observed that the HapU mutant Dd2 + M343L *pfcrt* clones showed step-wise increases in PSA and AUC levels with increasing *pm2/3* copy numbers (Fig. 6, G, H, and K). Removing the *pfcrt* M343L mutation from these parasites yielded a significant reduction in PSA and AUC levels (Fig. 6, G, H, and L). Our data also provided evidence that the amplification of *pm2/3* might constitute a first step of resistance as shown by enhanced parasite survival at lower PPQ concentrations (up to 100 nM). In the presence of multicopy *pm2/3*, the *pfcrt* M343L mutation further boosted parasite survival upon exposure to higher PPQ concentrations, and generated a biphasic response (Fig. 6, H and K). Our results reveal an epistatic genetic interaction between *pfcrt* and *pm2/3* in modulating levels of PPQ resistance.

### Multicopy *pm2/3* and mutant *k13* associate with reduced fitness in the RF7 KEL1/PLA1/PfPailin parasite

**Fig. 7.**
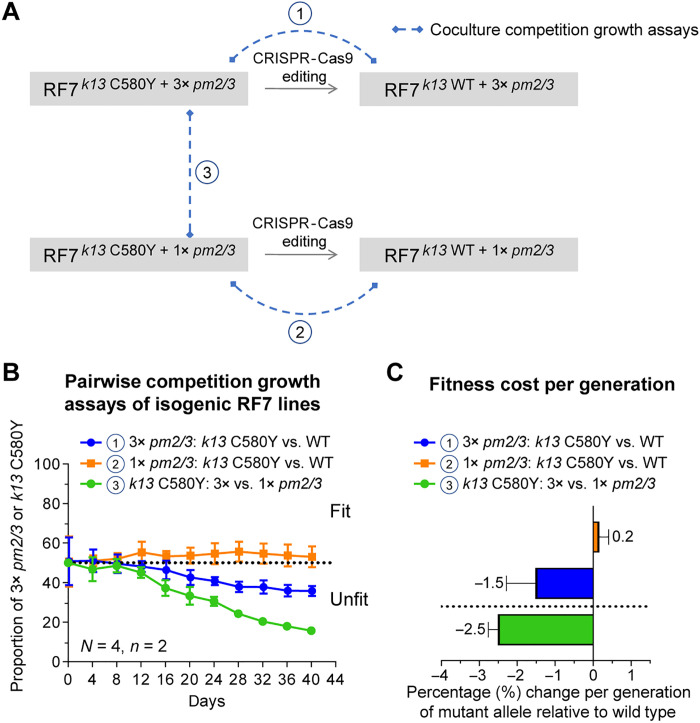
Impact of mutant *k13* and multicopy *pm2/3* on parasite asexual fitness. (**A**) Experimental design showing the generation of the panel of isogenic RF7 lines used in the coculture competitive fitness assays. (**B**) Pairwise competitive growth assays showing the proportion of *k13* C580Y or WT alleles in the RF7 line harboring either three copies or one copy of *pm2/3* and the proportion of RF7 lines carrying either three copies or one copy of *pm2/3*. Values shown are the averaged percentages from four independent experiments with two technical replicates per pairwise competition assay. (**C**) Fitness cost per generation, showing the relative change in percentage of the *k13* and *pm2/3* genotypes for each pairwise comparison.

We next investigated the impact of *k13* and *pm2/3* resistance determinants on parasite fitness in the absence of drug pressure. To study this, we performed long-term pairwise competitive assays between *k13* C580Y mutant versus WT clones that were edited in isogenic RF7 clones harboring one or three copies of *pm2/3* (Fig. 7A). The *k13* C580Y mutation exhibited a small fitness cost in the presence of multicopy *pm2/3*, whereas this k13 mutation was fitness neutral on the single-copy *pm2/3* background (Fig. 7, B and C). A greater fitness cost was observed with multicopy *pm2/3* compared to single-copy *pm2/3*, in *k13* C580Y RF7 parasites (Fig. 7, B and C). These data are consistent with our observation of the reduction of *pm2/3* copies in RF7 and HapU bulk parasites in the absence of selection pressure (fig. S8).

### The Dd2 *pfcrt* allele increases parasite susceptibility to the ACT partner drugs LMF and MFQ

**Fig. 8.**
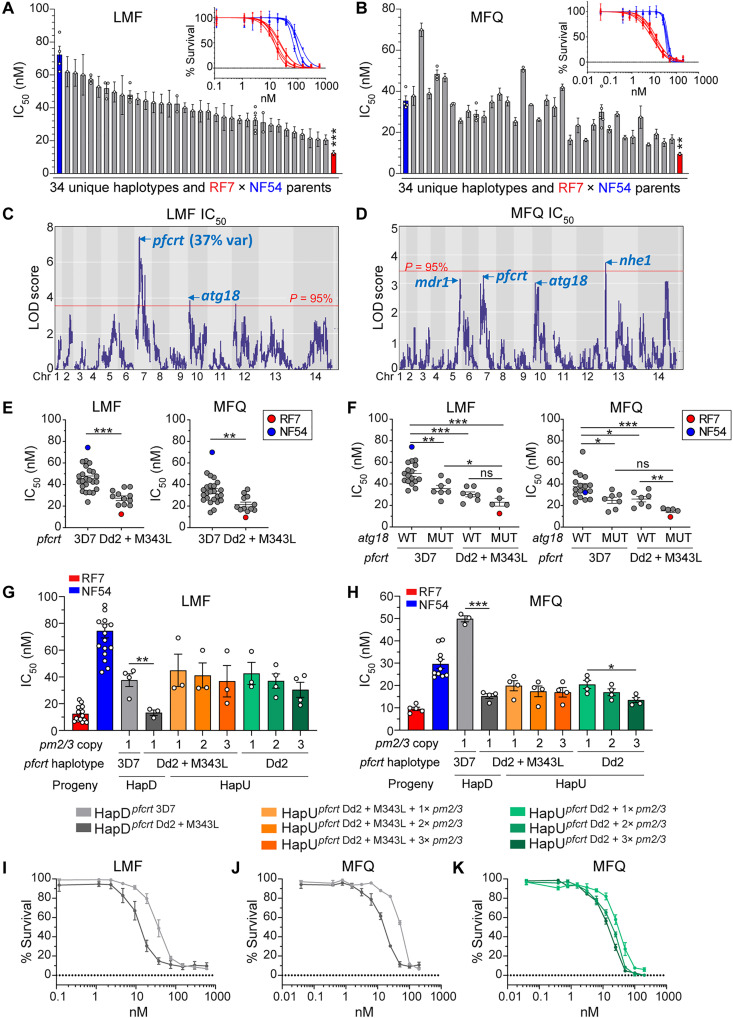
QTL mapping of LMF and MFQ response identifies multiple peaks including mutant Dd2 + M343L *pfcrt*. (**A**) LMF IC_50_ and (**B**) MFQ IC_50_ values of the 34 unique recombinant haplotypes and two parents. Each bar represents the mean ± SE IC_50_ for a recombinant haplotype (*N =* 4, *n* = 2). Haplotypes were ordered by LMF IC_50_ levels. For certain haplotype groups in which identical clones were obtained, multiple points depict data for more than one sibling progeny. Insets depict LMF and MFQ dose-response curves for RF7 (red) or NF54 (blue). Each curve represents the mean ± SD for an independent experiment. (**C** and **D**) LOD plots showing QTLs for LMF and MFQ above or near the 95% probability threshold (red line). (**E** and **F**) LMF and MFQ IC_50_ values for the 34 recombinant progeny and two parents, grouped by their *pfcrt* haplotypes (E) or grouped by their *atg18* and *pfcrt* haplotypes (F). MUT, mutant T38I. (**G**) LMF IC_50_ and (**H**) MFQ IC_50_ values in *pfcrt*-edited HapD progeny (3D7 versus Dd2 + M343L) having single *pm2/3* copy, and in edited HapU progeny (Dd2 versus Dd2 + M343L) having either one, two, or three copies of *pm2/3*. Bars represent the means ± SE (*N* = 3 to 4, *n* = 2). The colored key for parasite lines applies to (G) to (K). (**I**) LMF and (**J**) MFQ dose-response curves in edited HapD progeny showing that mutant Dd2 + M343L *pfcrt* confers increased tolerance to these drugs. Each line depicts the mean ± SE percent parasite survival (*N* = 3 to 4, *n* = 2). (**K**) MFQ dose-response curve in *pfcrt*-edited HapU progeny with variable *pm2/3* copies, showing that single *pm2/3* copy associates with reduced MFQ sensitivity. Each line depicts the mean ± SE percent parasite survival (*N* = 4, *n* = 2). Statistical significance was determined by Mann-Whitney *U* tests (A, B, E, and F) or unpaired Student’s *t* tests (G and H). *P < 0.05, **P < 0.01, ***P < 0.001.

Further profiling of the genetic cross parents revealed that RF7 has a three- to sixfold higher sensitivity to both LMF and MFQ compared with NF54 (Figs. 1B and 8, A and B). Phenotypic characterization of the progeny revealed a positive correlation between the IC_50_ values for both compounds (*R* = 0.66), indicative of similar although not identical mechanisms of resistance (Fig. 8, A and B, and fig. S4). This contrasted with the negative correlations (in the range of −0.35 to −0.45) observed between LMF or MFQ IC_50_ responses and either PPQ IC_50_ or AUC levels among the progeny (fig. S4). This observation is consistent with different markers of resistance and/or opposing alleles for the same set of markers. QTL analyses for LMF IC_50_ values revealed a dominant peak on chr7 within 11 kb of *pfcrt* (Fig. 8C and table S11). For MFQ, we observed multiple QTL peaks (including the same chr7 segment), suggesting a multigenic trait (Fig. 8D). When controlling for *pm2/3* as a covariate, we observed an increased association between the chr7 *pfcrt* QTL and MFQ IC_50_ values (fig. S9). Analysis of the IC_50_ values and *pfcrt* genotypes of the recombinant progeny revealed an association between mutant *pfcrt* and lower IC_50_ values for both LMF and MFQ (Fig. 8E).

To identify secondary markers of LMF and MFQ susceptibility, we performed a QTL analysis for LMF IC_50_ controlling for *pfcrt.* These data revealed significant QTLs on chr2 for LMF and chr10 for LMF and MFQ (fig. S9A and table S11). These segments contain *atg11* and *atg18*, respectively. Notably, the *atg18* T38I mutation (inherited from the RF7 parent) was associated with lower LMF and MFQ IC_50_ values in both WT and mutant *pfcrt* progeny (Fig. 8F). Progeny harboring both mutant *atg18* and mutant *pfcrt* were the most sensitive to these two drugs, whereas the double WT haplotypes were the least sensitive (Fig. 8F).

We next tested whether *pfcrt* might directly affect LMF and MFQ responses by assaying our *pfcrt*-edited isogenic progeny (Fig. 8, G and H). HapD progeny engineered to carry the mutant Dd2 + M343L *pfcrt* allele showed a significant threefold reduction in LMF and MFQ IC_50_ values compared with the WT *pfcrt* counterpart (Fig. 8, G to J). However, the *pfcrt* Dd2 + M343L variant showed no change in its LMF and MFQ IC_50_ values when compared to the Dd2 isogenic comparator (Fig. 8, G and H), suggesting that the eight–amino acid Dd2 *pfcrt* variant allele is solely responsible for heightened sensitivity to LMF and MFQ. We note a small but significant decrease in MFQ IC_50_ with higher *pm2/3* copies in the HapU *pfcrt*-edited progeny (Fig. 8, H and K).

### RF7 and NF54 exhibit differential susceptibility to preclinical compounds

**Fig. 9.**
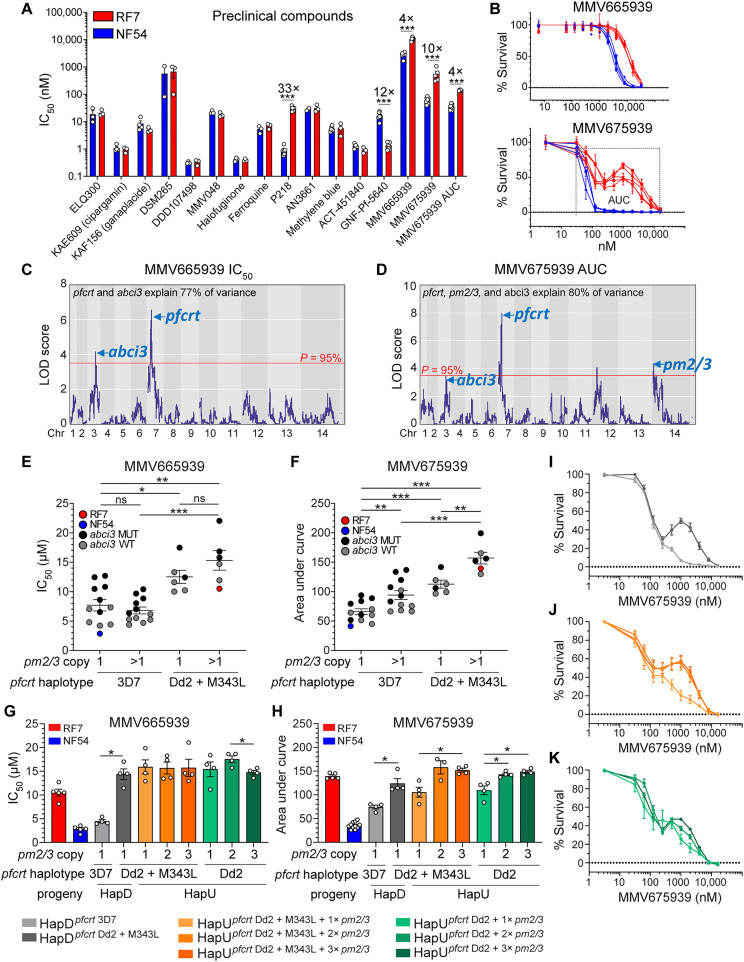
Phenotypic response of genetic cross parents, RF7 × NF54, to preclinical compounds and identification of *pfcrt*, *abci3*, *pm2/3* as markers of MMV665939 and MMV675939 resistance. (**A**) Seventy-two–hour drug susceptibility IC_50_ values for RF7 × NF54. IC_50_ fold shifts are indicated above the bars. Values represent the means ± SE (*N =* 3 to 7, *n* = 2). (**B**) Dose-response curves, showing a sigmoidal survival curve for MMV665939 and a biphasic response for MMV675939 in RF7. Each line represents the mean ± SD percent parasite survival per experiment (*N* = 4, *n* = 2). (**C** and **D**) LOD plots for MMV665939 IC_50_ and MMV675939 AUC levels showing QTLs above the 95% confidence threshold. (**E**) MMV665939 IC_50_ and (**F**) MMV675939 AUC levels for the 34 recombinant progeny and two parents, grouped by *pm2/3* copy, *pfcrt*, and *abci3* genotypes. (**G**) MMV665939 IC_50_ and (**H**) MMV675939 AUC levels in *pfcrt*-edited HapD progeny (3D7 versus Dd2 + M343L) and in edited HapU progeny (Dd2 versus Dd2 + M343L) having either one, two, or three copies of *pm2/3*. Values represent the means ± SE (*N* = 4, *n* = 2). The colored key for parasite lines applies to (G) to (K). (**I**) MMV675939 dose-response curves in unedited HapD (3D7) versus edited HapD (Dd2 + M343L) progeny, showing that mutant *pfcrt* is sufficient to generate a biphasic curve on a single-copy *pm2/3* background. For (I) to (K), each line depicts the mean ± SE percent parasite survival (*N* = 4, *n* = 2). (**J**) MMV675939 dose-response curves of HapU (Dd2 + M343L) with one to three copies of *pm2/3*, showing that multicopy *pm2/3* can augment the biphasic response. (**K**) MMV675939 dose-response curves in *pfcrt*-edited HapU (Dd2) progeny with one to three copies of *pm2/3*, showing that the M343L mutation is not required to generate a biphasic response on a Dd2 *pfcrt* background. Statistical significance was determined by unpaired Student’s *t* tests adjusted by multiple testing (A) or Mann-Whitney *U* tests (E to H). *P < 0.05, **P < 0.01, ***P < 0.001.

We tested the RF7 and NF54 response to a panel of 15 clinical and preclinical antimalarial compounds to map modulators of parasite susceptibility (Fig. 9A). Three of these compounds showed higher IC_50_ values in RF7. These include the antifolate P218 (*47*), which targets *P. falciparum* DHFR that in the case of RF7 is a quadruple mutant that mediates high-level resistance to the related antifolate PYM (*2*). Four- to 10-fold higher IC_50_ values were also observed for MMV665939 and MMV675939 (labeled collectively as “MMV6x”). In vitro resistance to the MMV6x5939 compounds was previously associated with single point mutations or CNVs in *abci3* (*48*, *49*). However, none of these resistance-conferring *abci3* mutations or CNVs were present in RF7 (fig. S10A). Nonetheless, the two *abci3* point mutations S2106C/S2227G in RF7 showed evidence of selection in field isolates (fig. S10B). This double mutant was present in ~37% of Cambodian or Thai isolates (*n* = 718) based on our analysis of 2512 isolates sequenced from Asia and Africa (*50*).

#### QTL mapping identifies pfcrt as a major determinant of a multigenic trait of reduced parasite susceptibility to MMV6x compounds

To map determinants of resistance to these MMV6x compounds, we phenotyped the cross progeny and performed QTL analysis (fig. S11A and Fig. 9B). This analysis showed that a narrow chr7 peak harboring *pfcrt* was the strongest predictor of MMV665939 IC_50_ and MMV675939 AUC levels (Fig. 9, C and D, and table S12). For both compounds, we also noted a smaller secondary QTL on chr3 containing the *abci3* locus. No other QTL peaks were observed for MMV665939 when controlling for *pfcrt* or *pm2/3* (fig. S9B). This observation suggests that MMV665939 resistance is associated with chr7 and chr3 segments, likely *pfcrt* and *abci3*. For MMV675939, we observed an additional peak on chr 14 that included *pm2/3* (Fig. 9D and fig. S9B). These data suggest that these three loci combine to underpin differential MMV675939 susceptibility.

#### Multicopy pm2/3, abci3, and pfcrt are combinatorial in modulating parasite susceptibility to MMV6x compounds

In our progeny, higher MMV665939 IC_50_ values and MMV675939 AUC levels were associated with both mutant *pfcrt* and *abci3* (Fig. 9, E and F). Higher MMV675939 AUC levels were also observed in progeny harboring multicopy *pm2/3* (Fig. 9F). For MMV675939, biphasic responses were observed either in progeny harboring mutant *pfcrt* or in progeny harboring mutant *abci3* in the presence of multicopy *pm2/3* (fig. S12). Gene editing of HapD by replacing WT with mutant *pfcrt* confirmed the role of this gene in MMV6x response (Fig. 9, G and H) and resulted in a biphasic MMV675939 dose response (Fig. 9I). Amplified *pm2/3* decreased parasite susceptibility to MMV675939 in HapU and HapU-edited lines, whereas MMV665939 was unaffected (Fig. 9, G and H). Genetically edited HapU parasites showed that the *pfcrt* M343L mutation had no impact on either MMV6x compound (Fig. 9, G and H). These data lead us to conclude that the Dd2 *pfcrt* allele itself can cause reduced susceptibility to MMV6x compounds and produce a biphasic response to MMV675939 (Fig. 9, J and K).

#### MMV6x compounds can interfere with mutant PfCRT-mediated efflux of PPQ and CQ

To investigate whether resistance to the two MMV compounds is attributable to PfCRT transport, we performed competitive inhibition assays to test their ability to inhibit PPQ or CQ uptake using proteoliposomes loaded with PPQ- or CQ-resistant mutant PfCRT isoforms, respectively (*32*). Control assays showed that CQ substantially inhibited PPQ uptake and vice versa. Verapamil, a known CQ resistance reversal agent, partially inhibited CQ and PPQ uptake. These results are consistent with earlier findings that verapamil can reverse resistance to both drugs via competitive inhibition of PfCRT-mediated drug transport (*27*, *36*, *51*). MMV675939 and MMV665939 both competed with PPQ and CQ for mutant PfCRT-mediated drug uptake, suggesting that these compounds can interact directly with PfCRT (fig. S11, B and C).

## DISCUSSION

By leveraging a *P. falciparum* genetic cross, we identified parasite loci associated with resistance to multiple first-line antimalarial drugs. Our studies of ART resistance identify two unlinked chromosomal segments that appear to contribute to ART resistance, in addition to mutant *k13* that our genotype-phenotype mapping localized as the primary determinant. We also report that PPQ resistance is dictated by an epistatic relationship between mutant *pfcrt* and multicopy *pm2/3*, which together achieve high-level resistance. However, this level of resistance comes with a fitness cost that leaves them vulnerable to replacement with WT *pfcrt* or single-copy *pm2/3* in the absence of PPQ pressure. We also show that the first-line partner drug LMF and its related arylaminoalcohol MFQ select against mutant *pfcrt*, underscoring a strategic advantage of implementing PPQ with either LMF or MFQ in the same field settings. Last, our data suggest that mutations in *abci3* form a network with *pfcrt* and multicopy *pm2/3* in regulating parasite susceptibility to diverse chemotypes, as illustrated with the two antiplasmodial drug discovery candidates MMV665939 and MMV675939.

Recent reports that *k13* mutations have emerged in several sites in east Africa and the Horn of Africa are a cause for concern, as reduced ART efficacy increases selective pressure on the partner drug and raises the prospects of future treatment failures (*52*). Intravenous artesunate is also first-line treatment for severe malaria (*53*), with mutant *k13*-mediated delayed parasite clearance potentially compromising treatment efficacy. Mutant *k13* parasites in the GMS are often more ART resistant and display improved fitness compared with African strains, suggesting that Asian strains have acquired secondary determinants (*12*, *22*). Our genetic cross data identify two candidate loci—a chr1 segment harboring *mrp1* and a chr14 segment harboring *arps10*. The four *mrp1* mutations present in RF7 (H191Y, S437A, I876V, and F1390I) are quite prevalent in the GMS and have been associated with altered susceptibility to ART derivatives (*54*, *55*). The most common of these mutations in Africa, I876V, was earlier associated with parasite recrudescence following artemether-LMF treatment (*56*). This transporter is nonessential in *P. falciparum* ABS parasites cultured in vitro, with knockout parasites showing increased parasite susceptibility to ART (*57*). In our genetic cross progeny, mutant *mrp1* was only found in parasites harboring mutant *k13*.

Our *arps10* marker on chr14, which harbors the V127M + D128H mutations, has been previously associated with prolonged parasite clearance half-lives following artesunate treatment and with increased RSA survival in K13 mutant parasites (*58*, *59*). This gene is thought to be a key founder mutation of the KEL1/PLA1 lineage on which ART resistance emerged in the GMS (*58*). Other mutations in this chr14 segment include exonuclease-V R25K and the alpha/beta hydrolase I301F, which were earlier identified as potential markers of DHA-PPQ treatment failures in Cambodian isolates (*24*). It is noteworthy that this segment overlaps the QTL associated with enhanced fitness from separate genetic cross studies with GMS parasites (*60*). These genes on chr 1 and 14 merit further examination as potential contributors to ART resistance emerging in African settings.

Our PPQ studies showed unexpected connections to ART resistance. Upon applying PPQ or CQ pressure to bulk progeny, we recovered resistant *pfcrt*-mutant progeny which were all mutant *k13*. PPQ also selected for the ART-resistant chr14 segment. These observations suggest that the selection of *pfcrt* mutants by PPQ in the field might have evolved on the background of mutant *k13* and favored the inheritance of the chr14 segment that might augment DHA resistance. Our results provide support that PPQ may have contributed to the dominance of the combined KEL1/PLA1 (*k13* C580Y and multicopy *pm2/3*) colineage across the GMS where DHA-PPQ was commonly used (*28*). The interconnection between PPQ and ART resistance presumably centers on heme, which serves as a target for PPQ and an activator for ART (*35*).

Our PPQ studies resolve debate about the relative roles of mutant *pfcrt* and multicopy *pm2/3* in driving resistance to this drug (fig. S13) (*27*, *28*, *37*). At PPQ concentrations <100 nM, we observed a decrease in parasite susceptibility upon amplification of *pm2/3* in isogenic progeny expressing either mutant Dd2 + M343L or Dd2 PfCRT isoforms. At 200 nM PPQ or above, the Dd2 + M343L variant afforded a clear gain of PPQ resistance, which increased with increasing *pm2/3* copy number. The biphasic nature of the PPQ dose-response curve was most pronounced with multicopy *pm2/3* on a mutant Dd2 + M343L *pfcrt* background.

The PPQ C_max_ in plasma is ~200 nM (*61*), which, based on our quantitative phenotypic analysis, would be consistent with both determinants contributing to resistance. The long terminal half-life of PPQ (~3 weeks) would also explain a selection advantage for multicopy *pm2/3*, which we found to enhance parasite survival at low PPQ concentrations. This partial gain of resistance likely favored the emergence of novel PfCRT variants that dominate PPQ resistance at higher drug concentrations (*62*). Biophysical studies using proteoliposomes attribute this to a mutant PfCRT-mediated gain of PPQ efflux away from its heme target in the DV (*32*). PfCRT-mediated PPQ or CQ efflux has also been shown using heterologous *Xenopus* oocyte or yeast assays (*33*, *36*, *63*, *64*).

PPQ and CQ appear to occupy partially overlapping sites in PfCRT, with some mutations including M343L contributing to PPQ resistance while becoming less CQ resistant (*27*, *32*, *36*). Notably, there is no established method to directly measure the impact of PfCRT isoforms on drug transport across the DV in parasites cultured in vitro, as was achieved earlier with PfMDR1 using fluorogenic substrates (*65*). For *pm2/3*, the basis for resistance might relate to its impact on the rate of hemoglobin proteolysis or conversion of heme to hemozoin, effectively depleting levels of heme that act as a ligand for PPQ binding. The earlier observation that *pm2* and *pm3* form part of a multicomponent hemozoin formation complex (*66*) may explain why amplification of both tandem genes is associated with PPQ resistance, yet overexpression of each gene individually did not affect the PPQ dose-response profile (*67*).

Our studies revealed that multicopy *pm2/3* exerts a substantial fitness cost in the Cambodian RF7 *k13* C580Y parasite, exceeding that observed with the *k13* mutation itself. We also observed spontaneous deamplification of *pm2/3* in parasites cultured for ~3 months in the absence of PPQ pressure. PPQ resistance–associated mutant PfCRT haplotypes are also known to exert a fitness cost (*27*, *68*, *69*). This growth defect has been attributed to an excessive buildup in the DV of globin-derived short peptides that serve as a natural substrate for PfCRT, thereby restricting protein synthesis (*70*–*72*). These observations help explain why the prevalence of both multicopy *pm2/3* and PPQ-resistant mutant *pfcrt* decreased rapidly following withdrawal of DHA-PPQ from first-line use in Cambodia (*62*).

A recent field-based and genetic cross study provided evidence for coselection of mutations in PfCRT and the putative DV-resident amino acid transporter PfAAT1 (*43*). RF7 harbors the S258L plus F313S PfAAT1 variant that was selected under CQ pressure in this previous cross (between NF54 and NHP4026). Our bulk segregant analysis of CQ-pressured bulk progeny pools also showed evidence of a minor peak at the *pfaat1* locus, in addition to strong selection for mutant *pfcrt*. Similar QTL studies with our progeny clones did not identify *pfaat1* under either CQ or no drug selection. We did, however, observe a peak for this segment in PPQ-pressured progeny. Further studies are clearly warranted to define the role of *pfaat1* in *P. falciparum* resistance to PPQ and CQ across different genetic backgrounds.

In some regions of the GMS, DHA-PPQ has been replaced by artemether-LMF, which to date has remained effective as LMF has not encountered resistance. Artesunate-MFQ has also been reintroduced into the GMS. Our QTL analysis data show a strong association between LMF or MFQ IC_50_ values and *pfcrt*, with these drugs selecting against the Southeast Asian mutant Dd2 + M343L allele. We confirmed these results in *pfcrt* gene-edited parasites. These data provide evidence that LMF and PPQ can exert opposing selective pressures on the PfCRT locus, in agreement with earlier observations that LMF selects against CQ-resistant *pfcrt* as detected using the K76T marker (*31*, *73*).

In Africa, artemether-LMF accounts for 75% of the antimalarial drug market (*1*). Recent studies have shown excellent efficacy of DHA-PPQ either for intermittent preventive treatment of infants or pregnant women or for seasonal malaria chemoprevention (*74*). Our data lend support to the introduction of DHA-PPQ into areas with artemether-LMF use. Clinical teams have also documented excellent clinical efficacy of triple therapies that combine DHA-PPQ with MFQ in the GMS, with the rationale that use of these triple ACTs can suppress the emergence of multidrug resistance (*75*). We observed a slight sensitization of our multicopy *pm2/3* progeny toward MFQ, a drug that is known to select for multicopy *pfmdr1.* However, no association was observed with LMF. These data may help explain why *P. falciparum* parasites in the GMS very rarely show amplification of both *pfmdr1* and *pm2/3* (*62*).

Our genetic mapping of candidate loci is constrained by the choice of resistant parent and the resolution of the meiotic crossover events. Nonetheless, our data illustrate the power of genetic crosses for identifying polygenic networks of *P. falciparum* multidrug resistance. We note that two separate genetic crosses in the humanized mouse model were recently reported with Southeast Asian parasites harboring the ART-resistant *k13* C580Y mutation (*41*). Bulk segregant analysis of DHA-treated synchronized progeny pools only identified the *k13* segment on chr13. In our study, we profiled the DHA response (using RSA and AUC) for each recombinant progeny and parent and uncovered distinct peaks in addition to *k13*. For PPQ, our QTL analysis on individual progeny identified *pm2/3* as a second peak that was not observed in the bulk segregant analysis of PPQ versus untreated bulk pools. These data provide evidence that bulk segregant analysis is effective at identifying primary determinants, but QTL analyses of individual progeny can provide more granular resolution of complex genetic traits and uncover epistatic interactions.

Crosses provide an important complement to genome-wide association studies as well as in vitro evolution and WGS studies that can identify candidate chromosomal segments and specific loci (*23*, *24*, *48*, *76*). These approaches will be key to identifying the genetic basis of *P. falciparum* resistance to first-line combination therapies, which appears to be imminent in Africa, and leveraging that knowledge to map the spread of resistance and develop strategies to mitigate its impact.

## MATERIALS AND METHODS

### *P. falciparum* infections in *A. stephensi* mosquitoes and humanized chimeric liver mice

#### Parasite lines, gametocyte cultures, and mosquito feeding

The RF7 [Sanger ID: PH1008-C (27)] Cambodian isolate was provided by R. Fairhurst and was a recrudescent sample of PH1356-C (genetically identical except that the latter had only a single copy of *pm2/3*). The NF54 isolate was provided by P. Sinnis. RF7 and NF54 gametocytes were cultured as previously described (*77*). Briefly, parasites were grown in RPMI 1640 containing 10% (v/v) human serum and 4% red blood cell (RBC) hematocrit at 37°C in a glass candle jar. Cultures were initiated at 0.5% asynchronous asexual parasitemia from a low-passage stock and maintained up to day 18 with daily media changes but without any addition of fresh erythrocytes. Day 15 to 18 cultures, containing largely mature gametocytes, were used for mosquito feeds. Cultures were centrifuged (108*g*, 4 min), and the parasite pellet was resuspended to 0.6% gametocytemia in a mixture of human O^+^ RBCs supplemented with 50% (v/v) human serum. To genetically cross RF7 and NF54, we used a 1:1 ratio of gametocytes at a final gametocytemia of 0.6%. Gametocytes were fed to 2- to 4-day-old *A. stephensi* mosquitoes (Liston strain) that had been starved overnight, using glass membrane feeders. Unfed mosquitoes were removed after feeding. Fed mosquitoes were then maintained on a 10% sugar solution at 25°C and 80% humidity with a 14-hour:10-hour (light:dark) cycle including a 2-hour dawn/dusk transition. Human RBCs were collected weekly from healthy volunteers, with informed consent, under the Johns Hopkins University Institutional Review Board approved protocol NA_00019050. All experiments were performed in accordance with institutional guidelines and regulations.

#### Oocyst and salivary gland sporozoite quantification

On day 7 postinfected blood meal, mosquito midguts were harvested and stained with mercurochrome. Oocysts were counted by brightfield microscopy using a Nikon E600 microscope with a PlanApo 10× objective. We observed a 75% prevalence of infection, with an average of 10.7 oocysts per mosquito. Between days 14 and 16, salivary glands were harvested from a minimum of 20 mosquitoes and homogenized, and the sporozoites were counted using a hemocytometer. On average, we obtained 25,000 salivary gland sporozoites per mosquito.

#### Mouse infections

All animal experiments were performed in accordance with the Johns Hopkins University Animal Care and Use Committee guidelines (approved protocol M017H325). HuHep mice were purchased from Yecuris (FRG-NOD, human hepatocyte repopulated 70%+; catalog no. 10-0013). Upon arrival, we began withdrawal of the mice from 2-(2-nitro-4-trifluoromethylbenzoyl)-1,3-cyclohexanedione. Mice were kept in a sterile facility with sterile bedding, food, and water. Mice were weighed daily, and those that showed any weight loss >10% (compared to preshipment weight) were treated with 300 μl of sterile saline via intraperitoneal injection and given a nutritional supplement in water (STAT; https://prnpharmacal.com/products/nutritional-supplements/stat/). Mice were allowed to recover from shipping for 2 to 3 days before infection. Infection with *P. falciparum* sporozoites was performed either by mosquito bite or by intravenous inoculation. For the mosquito bite approach, mosquitoes were allowed to probe/feed on the isoflurane-anesthetized mice for 15 to 20 min. Each of the three mice received approximately 50 to 60 infectious mosquito bites. A fourth mouse was intravenous-inoculated with 3 million sporozoites dissected from mosquito salivary glands. Both routes were chosen on the basis of earlier reports of successfully using either intravenous injection or mosquito bites (*39*, *40*). Mice infected by mosquito bite remained stable with a healthy appearance for the remainder of the experiment. In contrast, the intravenous-inoculated mouse lost >10% body weight and on day 4 post-inoculation appeared ill. This latter mouse was given 3000 U of penicillin via the intramuscular route, 1000 U of penicillin and 1 mg of streptomycin intraperitoneally, and additional daily doses of penicillin and streptomycin until sacrifice. On days 6 and 7 post-sporozoite infection, 500 μl of RPMI-washed human RBCs was inoculated intravenously into each mouse. At day 7.5 postinfection, ABS parasites were detected at up to 0.3% parasitemia, and blood was recovered by cardiac puncture of mice anesthetized with ketamine/xylazine. One mouse, M46056, which received 3 million sporozoites via the intravenous route, showed the highest parasitemia on day 7 and was used for bulk selection experiments (table S2).

### In vitro *P. falciparum* cell culture

*P. falciparum* ABS parasites were cultured in human O^+^ RBCs obtained from the Interstate Blood Bank (Memphis, TN) at 3% hematocrit, using RPMI 1640 medium supplemented with 25 mM Hepes, sodium bicarbonate (2.1 g/liter), gentamicin (10 μg/ml), 50 μM hypoxanthine, 0.5% (w/v) AlbuMAX II (Thermo Fisher Scientific), and 10% (v/v) human serum (Interstate Blood Bank). Parasites were maintained at 37°C under 5% O_2_/5% CO_2_/90% N_2_ gas conditions. Single clones were obtained from the bulk progeny pools, the RF7 parent, and the HapU progeny by limiting dilution cloning at 0.5 parasites per well. Culture medium was replenished twice a week, and fresh RBCs were added once per week. Clones were expanded from days 19 to 35 after parasitemias were above the detection threshold, and the *pm2* copy number was measured by TaqMan-based real-time polymerase chain reaction (PCR) on isolated genomic DNA (gDNA; see the section below). Protocol AAAU3761, describing the use of human RBCs from anonymized, de-identified donors for *P. falciparum* cultures, was approved by the Columbia University Institutional Review Board, who deemed this to be not human subjects research under 45 CFR 46.

### Drug treatments applied on bulk progeny pools

We applied 75 nM PPQ (5.6× IC_50_ for NF54), 200 nM mdCQ (9.1× IC_50_ for NF54), and 1 μM PYM (54× IC_50_ for NF54) for 72 to 96 hours on bulk progeny pools in 10-ml culture volumes at 3% hematocrit and ~1% parasitemia. Drug was replenished every 24 hours with media changes. Following 72 or 96 hours of drug exposure, drug removal was achieved through centrifugation of the culture at 1500 rpm for 5 min, discarding the supernatant and resuspending the cultures in 50 ml of fresh media, with three washes in total. Cultures were monitored daily and harvested for gDNA once parasitemia reached at least 1%, which was 8 days from the start of treatment for PYM and mdCQ and 16 days for PPQ. On the basis of our initial tests, we found that these drug exposure conditions effectively cleared NF54 but not RF7 parasites and thus proved suitable to eliminate selfed NF54 progeny, thereby increasing the numbers of independent recombinant progeny. We did not subject these bulk progeny pools to DHA pressure because the other drug selections already yielded progeny with diverse DHA phenotypes, as measured using RSA and AUC values.

### WGS of bulk pools and individual progeny

Parasite cultures at 1 to 5% parasitemia were lysed with 0.1% saponin in 1× phosphate-buffered saline (PBS), and gDNA was then extracted using the QIAamp DNA Blood Mini Kit (Qiagen). DNA concentrations were determined using the Qubit dsDNA BR assay kit (Thermo Fisher Scientific). WGS libraries were prepared using the Illumina Nextera DNA Flex protocol. Briefly, 150 ng of gDNA was fragmented and tagmented using bead-linked transposons and subsequently amplified by 5 cycles of PCR to add dual-index adapter sequences to the DNA fragments. The libraries were quantified, pooled, and sequenced on Illumina MiSeq or NextSeq platforms to generate 300- or 150-bp paired-end reads, respectively. Drug-selected bulk cultures yielded a mean 44× depth of coverage by Illumina MiSeq sequencing. Cloned progeny were subjected to WGS by Illumina NextSeq or MiSeq to identify selfed versus genetic recombinants. Thereafter, representative progeny from each of the 34 recombinant haplotypes, as well as the two parents RF7 and NF54, were resequenced on the Illumina MiSeq platform to obtain between 2.7 and 4.6 M reads, corresponding to an average of 40.7× depth of coverage, with on average at least 94.1% of each genome having 10 or more reads (table S3). Sequence data were aligned to the *P. falciparum* 3D7/NF54 genome (PlasmoDB version 36.0) using Burrow-Wheeler Alignment. Reads that did not map to the reference genome, as well as optical duplicates, were removed using SAMtools and Picard. The reads were realigned around indels using Genome Analyses Tool Kit (GATK) RealignerTargetCreator, and base quality scores were recalibrated using GATK BaseRecalibrator.

All variants were called using *mpileup* and were filtered on the basis of quality scores (minimum base quality score ≥ 20, mapping quality > 30, read depth ≥ 5) and multiallelic = FALSE to obtain high-quality SNPs, which were then annotated with snpEFF. SNPs for highly polymorphic surface antigens and multigene families (notably *var*, *stevor*, and *rifin*, located mostly at the subtelomeric ends of chromosomes) were excluded as these are prone to stochastic changes during in vitro culture. To obtain the list of SNPs that differed between the parents in the core genome, we retained only homozygous SNPs, based on >90% alternate allele frequency in RF7 and >90% reference allele frequency in NF54. For QTL mapping, *pm2/3* copy was manually imputed as an additional marker “Pf3D7_14_v3_295261_PM2.” This analysis gave a total of 18,490 SNPs (18k SNP set). SAMTools mpileup was used to find SNPs in the 18k SNP set for progeny or in targeted genomic regions including genes mediating known drug resistance phenotypes. SNPs that showed an allele frequency between 10 and 90% in the progeny clones were classified as heterozygous and defined as “missing/undetermined.” Bayesian information criterion (BIC)-Seq was used to check for copy number variations using the Bayesian statistical model (*78*). Copy number differences between RF7 and NF54 were only observed in a chr14 segment harboring the tandem *pm2/3* genes.

### Microsatellites for identifying recombinant progeny and for determining PfPailin genotypes within the *k13*-flanking regions

To provide a microsatellite (MS) signature for each recombinant progeny, we established a list of MS markers that differentiated RF7 from NF54. For this analysis, we wrote a custom Python script, using the pysam module, to call WGS reads showing insertions or deletions at targeted genomic loci (*79*). Integrated Genome Viewer was used to visually verify the presence of MS markers. The markers used herein were C2M18, TAA81, BM5, C3M67, TA1, and C13M87 (fig. S14). To identify MS markers surrounding the *k13* gene, we referred to markers previously profiled in PfPailin parasites that spanned −50.0 to +31.5 kb of this gene (*5*, *80*) and determined these marker sizes in the progeny based on WGS reads (fig. S3).

### Confirmation of progeny clones via MS-based multiplexed fragment analysis

We used multiplexed fragment analysis to validate the genetic purity of individual progeny clones, pre- and postphenotyping (fig. S14). PCRs were set up individually for C2M18, TAA81, BM5, C3M67, TA1, and C13M87, using forward primers fluorescently labeled with 6-FAM (blue), ATTO550/NED (yellow), or ATTO532/VIC (green) as described previously (*79*). PCR products treated with ExoSAP-IT Express Reagent (Thermo Fisher Scientific) were sent for capillary electrophoresis with the Liz500 ladder as reference (Genewiz). Data were analyzed using Peak Scanner 2 (Thermo Fisher Scientific) to determine MS sizes, with RF7 or NF54 included as references.

### Phenotypic assessment of drug sensitivities in genetic cross parents and progeny clones

#### Drug susceptibility assays for RF7 × NF54 parents

To profile the genetic cross parents’ susceptibility to a panel of 30 antimalarial drugs or preclinical compounds, we performed 72-hour dose-response assays on sorbitol-synchronized ring-stage cultures in 3 to 17 independent experiments with technical replicates. Assays (with 0.4 to 0.7% starting parasitemia and 1% hematocrit) used a 10-point twofold serial dilution of the compounds and included 0.1% dimethyl sulfoxide (DMSO) control-treated samples.

#### DHA and PSAs

To obtain tightly synchronized parasites for RSAs and PSAs, cultures were treated with 5% d-Sorbitol (Sigma-Aldrich) for 15 min at 37°C to remove mature parasites. For RSAs, we then cultured for 40 hours and purified late-stage segmented schizonts over 35 and 65% Percoll double density gradients (*81*). Purified schizonts were incubated with fresh RBCs for 3 hours and treated with 5% d-Sorbitol to obtain 0- to 3-hour postinvasion early rings. These rings were exposed to DHA concentrations ranging from 5.5 to 2,800 nM, prepared as twofold serial dilutions in 96-well plates. Wells were inoculated with 200-μl samples (at 1% starting parasitemia and 1% hematocrit), with technical duplicates. Control wells contained 0.1% DMSO. After a 4-hour incubation at 37°C, wells were washed three times with complete medium to remove drug, and contents were transferred to fresh 96-well plates using the Freedom EVO MCA96 liquid-handling instrument (Tecan). Cultures were subsequently maintained for an additional 66 hours in drug-free medium. For PSAs, sorbitol-synchronized ring-stage parasites were exposed for 72 hours to 200 nM PPQ (dissolved in 0.5% lactic acid), alongside drug-free wells, in technical duplicates.

#### PPQ, LMF, MFQ, MMV665939, and MMV675939 drug susceptibility assays

For the PPQ AUC measurements, we performed 72-hour assays with synchronized ring-stage parasites exposed to PPQ at twofold serially diluted concentrations ranging from 0.8 nM to 25.6 μM, alongside drug-free wells, in technical duplicates. Standardized 72-hour dose-response assays were conducted similarly for LMF, MFQ, MMV665939, and MMV675939 at 10-point concentrations starting from 600 nM, 200 nM, 32 μM, and 16 μM for these compounds, respectively. DMSO-treated control wells (0.1%) were run in parallel. For all drug assays, the RF7 and NF54 parents were included as reference controls.

#### Flow cytometry for quantification of viable parasites

Parasitemia for drug-treated and vehicle control–treated wells were measured at 72 hours by flow cytometry, as previously described (*15*). Briefly, parasites were incubated with 1× SYBR Green (Thermo Fisher Scientific) and 100 nM MitoTracker DeepRed (Thermo Fisher Scientific) for 30 min at 37°C, followed by quenching with 1× PBS. On average, we analyzed 10,000 cells per sample, using an iQue Screener Plus cytometer (Sartorius). Viable parasites were defined as the percentage of MitoTracker-positive– and SYBR Green-positive–labeled cells.

#### Calculation of RSA, PSA, AUC, and IC_50_ values

For all drug assays conducted here, we included kill controls in which 1 μM DHA-treated parasites were used as a background control to achieve complete parasite killing and subtracted this percent parasitemia from the parasitemias measured for each well. Parasite survival in the presence of DHA or PPQ was expressed as the percentage of the background-subtracted parasitemia in the 700 nM DHA-treated samples or 200 nM PPQ-treated samples divided by the parasitemia of the DMSO-treated samples. Mean RSA survival rates of >1% were defined as DHA resistant (*82*). The AUC values for DHA, PPQ, and MMV675939 were calculated on the basis of total parasite survival across the range of 21.9 nM to 2.8 μM for DHA, 3.1 nM to 25.6 μM for PPQ, and 31.3 nM to 16 μM for MMV675939. IC_50_ values were determined by applying a nonlinear regression model (sigmoidal dose-response with variable slope) on the normalized % survival across the log-transformed drug concentrations, using Prism v8.3.1 (GraphPad).

### Genetic map construction

Duplicate markers were removed to obtain 2,091 markers using R/qtl (*83*). These markers were analyzed in JoinMap v5 (*84*) using the independence LOD parameter to generate a single linkage group for each chromosome, except for chr12 where there were three linkage groups. Using the resulting 1918 markers, we constructed a genetic map for RF7 × NF54 using Kosambi’s regression mapping function and adjusted for linkages with a recombination frequency < 0.5 and LOD scores > 0 (table S6).

### Bulk segregant analysis of bulk pools and drug-selected clones

Identification of QTLs in bulk pools treated with each of the drug conditions was conducted on the 18k SNP set obtained from WGS data using G′ and Quantitative trait locus (QTL)-seq approaches in the QTLseqr package in R (*85*). A sliding window of 100 kb was used for calculating the tricube-smoothed G′ and ∆(SNP index) values of each SNP within that window. False discovery rate *q* values were adjusted and customized for each drug-to-drug or drug-to-control pairwise analysis, which enabled us to account for the differences in sequencing coverage and the varying degrees of loci enrichment between drug-selected bulk pools. Outlier SNPs were filtered by Hampel’s rule (*85*). In addition to the bulk segregant analysis conducted between drug-treated bulk pools (Fig. 2) and between drug-treated and untreated bulk pools (fig. S2), we performed a similar analysis on progeny clones from bulk progeny pools that were exposed to drug or derived in the absence of drug. For this analysis, progeny clones selected by PPQ, mdCQ, PYM, or untreated controls (*N* = 12 for untreated, *N* = 12 for PYM, *N* = 7 for mdCQ, and *N* = 6 for PPQ) were grouped, and the RF7 allele frequency within each group of clones was averaged, thereby generating pooled clones that was used for bulk segregant analysis (Fig. 3E).

### Individual clone-based quantitative trait loci mapping

After filtering out SNPs that were missing in >5 of the 49 progeny (representing the 34 haplotypes) profiled for drug susceptibilities, we retained 15,869 SNPs and used these with the R/qtl2 package (*86*) to map QTL peaks. To identify significant QTLs for each phenotypic output, permutations of phenotypic data were performed 1000 times to obtain a distribution of maximum LOD scores. These scores were then used to calculate the LOD threshold at 95% probability. Fine-mapping of the QTL segments was subsequently performed using Bayesian interval mapping at a 95% confidence level. To elucidate secondary QTLs, LOD scores were calculated after setting the major peak as an additive covariate. The LOD threshold at 95% probability was then recalculated. The *k13*, *pfcrt*, and *pm2/3* genes were applied separately as additive covariates for each of the QTL analyses. The percent variance associated with each QTL was determined by establishing linear models for each QTL and comparing additive versus interactive QTL effects in R/qtl (*83*).

### Gene editing of *pfcrt* and *k13* by ZFN-based and/or CRISPR-Cas9 approaches

We performed CRISPR-Cas9 editing to remove the C580Y mutation from the *k13* locus in RF7 clones harboring either one or three copies of *pm2/3*, using the all-in-one plasmid, pDC2-cam-coSpCas9-U6-gRNA-k13_bsm-hdhfr (*12*). We also used zinc-finger nuclease-mediated editing to replace the 3D7 WT *pfcrt* allele with the Dd2 + M343L *pfcrt* isoform (carrying nine point mutations) in HapD progeny, as previously described (*87*). CRISPR-Cas9 editing of *pfcrt* to remove the M343L single-point mutation in the HapU progeny harboring either 1, 2, or 3 copies of *pm2/3* was performed using the all-in-one plasmid, pDC2-cam-Cas9-U6-gRNA-pfcrt_M343-bsd. Plasmid cloning was performed as for *k13*, except that the guide RNA (gRNA) was inserted using In-Fusion cloning (Takara), and blasticidin S-deaminase (bsd) was used as the drug selection cassette instead of human *dhfr*. gRNAs were cloned using the primer pair p8234 + p8235. Donor templates were amplified and cloned into the final vector using the primer pairs p8236 + p8237. Site-directed mutagenesis was performed using the allele-specific primer pairs p8238 + p8239. Donor inserts were PCR-amplified using the primer pair p7949 + p7950 and sequenced with the internal primer p7989. The primer sequences are listed in table S13.

To generate gene-edited lines, ring-stage parasites at 5 to 10% parasitemia were electroporated with 50 μg of circular plasmid DNA resuspended in Cytomix. Transfected parasites were selected by culturing in the presence of WR99210 (Jacobus Pharmaceuticals) or blasticidin for 6 to 8 days post-electroporation. The RF7 clones were subjected to 5 nM WR99210, while the HapD and HapU parasites were selected under 2.5 nM WR99210 or blasticidin (2 μg/ml). Parasite cultures were monitored for recrudescence by microscopy for up 8 weeks post-electroporation. To screen for successful editing, the *k13* and *pfcrt* loci were amplified directly from parasite cultures using the MyTaq Blood-PCR Kit (Bioline) (*79*). PCR products were submitted for Sanger sequencing, and edited parasites were cloned by limiting dilution. Gene-edited lines and their phenotypes are described in table S8.

### Quantitative PCR for determination of *pm2* copy number

Multiplexed TaqMan quantitative PCR (qPCR) of *pm2* labeled with FAM and single-copy β*-tubulin* labeled with HEX (as an internal control) were performed on gDNAs extracted from the two parents, progeny clones, and gene-edited lines, pre- and postphenotyping. Reactions were run on a QuantStudio 3 real-time PCR system (Thermo Fisher Scientific) (*79*). Five standards of gene fragments, mixed at 1:1, 2:1, 3:1, 4:1, and 5:1 molar ratios of *pm2*:*β-tubulin*, as well as the 3D7 line (one copy of *pm2*), were included as copy number controls. Each sample was assayed in technical duplicates. The *pm2* copy number for each progeny line was calculated by normalizing to the 3D7 control, using the relative quantification method (*79*). Primer sequences are listed in table S13.

### Competitive fitness assays for *k13* and *pm2/3*

#### Isogenic cocultures

Fitness assays were performed by coculturing isogenic parasite lines in 1:1 ratios. These assays paired isogenic *k13* WT versus mutant clones in RF7 clones that harbored either one or three copies of *pm2/3.* We also paired single versus multicopy *pm2/3* RF7 clones. These three pairwise cocultures were used to independently examine *k13* genotype or *pm2/3* copy number. Assays were initiated with tightly synchronized rings and conducted on four independent occasions with technical replicates. Cultures were maintained at 6-ml volumes over a period of 40 days (~20 generations), and every 4 days a fraction of each coculture was harvested for gDNA as described above.

#### k13 allelic discrimination qPCR assays

The percentage of WT or mutant *k13* alleles in each sample was determined using custom TaqMan fluorescence-labeled minor groove binder (MGB) probes in TaqMan allelic discrimination real-time PCR assays, as described previously (*12*). Probes were designed to specifically detect either the *k13* C580Y propeller mutation (HEX probe, Eurofins) or the WT allele (FAM probe, Eurofins). Primer sequences are listed in table S13. Mixtures of WT and mutant plasmids in fixed ratios (0:100, 20:80, 40:60, 50:50, 60:40, 80:20, and 100:0) were run as parallel controls to ensure accurate allelic detection. qPCR reactions for each sample were run in duplicate, with each 20-μl reaction consisting of 1× QuantiFAST reaction mix containing ROX reference dye (Qiagen), 0.66 μM forward and reverse primers, 0.16 μM FAM-MGB and HEX-MGB TaqMan probes, and 10 ng of gDNA. Cycling conditions were as follows: 1 cycle of 30 s at 60°C and 5 min at 95°C and 40 cycles of 30 s at 95°C and 1 min at60°C. Every assay included no-template negative controls as well as positive controls (mixtures of WT and mutant plasmids in fixed ratios), which were run in triplicate. Amplification and detection of fluorescence were carried out on a QuantStudio3 real-time PCR system using the genotyping assay mode. Rn (the fluorescence of the FAM or HEX probe) was normalized to the fluorescence signal of the ROX reporter dye. Background-normalized fluorescence (Rn minus baseline or ΔRn) was calculated as a function of cycle number.

#### pm2/3 allelic discrimination qPCR assays

TaqMan allelic discrimination was not suitable to quantify the percentage of single versus two or more copies of *pm2/3* as the multicopy alleles have identical sequences. To facilitate the differentiation of RF7 clones expressing one or three copies of *pm2/*3, we therefore conducted WGS of *pm2/3* multicopy versus single-copy RF7 clones. This analysis identified a SNP (gaA/gaT) present within the isoleucine-tRNA ligase gene (Pf3D7_1332900), corresponding to an E459D mutation in the RF7 clone harboring a three-copy *pm2/3* amplicon, presumably as a result of genetic drift during extended culture and clonal expansion. Forward and reverse PCR primers and custom TaqMan fluorescence-labeled probes were designed to target the E459 WT (FAM, Eurofins) or E459D mutant (HEX, Eurofins) alleles for this gene (table S13).

The efficiency and sensitivity of these TaqMan primers were assessed using standard curves comprising 10-fold serially diluted templates ranging from 10 to 0.001 ng. Robustness was demonstrated by high efficiency (94 to 96%) and *R*^2^ values (0.99 to 1.00). The quantitative accuracy in genotype calling was assessed by performing multiplex qPCR assays using mixtures of WT and mutant plasmids in predefined fixed ratios (0:100, 20:80, 40:60, 50:50, 60:40, 80:20, and 100:0). These mixtures were obtained by cloning a ~800-bp fragment for isoleucine-tRNA ligase centered around the mutant or WT SNP in pGEM plasmids. Triplicate data points clustered tightly and gave expected ratios of WT to mutant alleles, indicating high reproducibility in the data across the fitted curve (*R*^2^ = 0.86 to 0.87). Cocultures consisting of the RF7 isogenic clones containing multicopy *pm2/3* with the isoleucine-tRNA ligase E459D mutation, and the single-copy *pm2/3* and WT E459 allele, were initiated at a 1:1 ratio, and fitness assays were conducted over a 40-day period. qPCR reactions were run with probes targeting the isoleucine tRNA ligase marker that was diagnostic for single versus multicopy *pm2/3*, using conditions described above.

To determine the WT or mutant allele frequency in each sample, we retained only ΔRn values in samples where the threshold cycle (*C*_t_) was at least 3 cycles less than the no-template control. Next, we subtracted the ΔRn of samples from the ΔRn of the no-template negative control. We subsequently normalized the fluorescence to 100% using the positive control plasmids (tested in parallel reactions) to obtain the percentage of the WT and mutant alleles for each sample. The final percentage of the mutant allele was defined as the average of two values: the normalized percentage of the mutant allele and 100% minus the normalized percentage of the WT allele.

#### Fitness cost assessment

The fitness cost associated with the *k13* mutation or multicopy *pm2/3* was calculated relative to its isogenic WT or single-copy counterpart, respectively, using the following equation: *P*′ = *P* × (1 − *f*)*^n^*. *P*′ is the percentage of line expressing the *k13* C580Y mutation or multicopy *pm2/3* at the assay endpoint. *P* is the percentage of the coculture that expresses the *k13* WT allele or single-copy *pm2/3* on day 0. *n* is the number of generations from the assay start to finish, and *f* is the fitness cost. For the TaqMan allelic discrimination real-time PCR assays described above, *n* was set to 20.

### CQ or PPQ uptake assays in PfCRT-containing proteoliposomes

Inhibition of CQ or PPQ uptake was measured using proteoliposomes containing PfCRT, as described previously (*32*) with the following modifications. Purified PfCRT variants were reconstituted in preformed liposomes made of *Escherichia coli* total lipids:cholesteryl hemisuccinate in a ratio of 97:3 (w/w), and the lumen of the proteoliposomes was composed of 100 mM KP_i_, (pH 7.5) and 2 mM β-mercaptoethanol. Uptake of 100 nM [^3^H]-CQ (1 Ci/mmol) or [^3^H]-PPQ (1 Ci/mmol) was performed by diluting PfCRT-containing proteoliposomes (30 ng of PfCRT per assay) in 50 μl of 100 mM tris/MES (pH 5.5) in the absence or presence of 1 μM atovaquone, CQ, PPQ, MMV665939, MMV675939, or verapamil. Valinomycin (1 μM) was added to the reaction to generate a K^+^ diffusion potential-driven membrane potential. After 30 s, the reactions were quenched by the addition of ice-cold 100 mM KP_i_ (pH 6.0) and 100 mM LiCl before filtration. The radioactivity retained on the filters was determined with scintillation counting in a Hidex SL300 scintillation counter. Proteoliposomes that contain the CQ-resistant 7G8 or the PPQ-resistant 7G8 + F145I or 7G8 + C350R PfCRT isoforms were used herein. No drug (“−”) and atovaquone were included as negative controls, for which we expected no or minimal inhibition of CQ or PPQ uptake. CQ or PPQ was used as the positive control as PPQ has been reported to interact with PfCRT and inhibit binding of CQ (*32*). Data were normalized to the specific signal (total counts per minute in PfCRT-containing proteoliposomes minus counts per minute in liposomes devoid of PfCRT). Means ± SE values are presented as the percentage of the uptake obtained in the absence of drug (“−”). Data were collected from three independent experiments with technical duplicates.

### Statistical analysis

Unless stated otherwise, experiments were performed on at least four independent occasions, and exact sample sizes are given in the figure legends or supplementary tables and figures. Scatterplots and bar graphs are presented as means ± SE. Data were analyzed and plotted using Prism v8.3.1 (GraphPad). QTL mapping was analyzed and plotted in R using R/qtl, R/qtl2, and ggplot2. Comparisons between two groups were analyzed by Mann-Whitney *U* tests if at least four repeats were obtained per group or unpaired Student’s *t* test if otherwise. Statistical significance was defined by two-sided *P* values (*P < 0.05, **P < 0.01, and ***P < 0.001). *P* values less than 0.05 were considered statistically significant. ns indicates no significant difference.

## Supplementary Material

20231108-1

## Notes

**Correction (14 November 2023):** Due to a production error, the units in the y axis labels in Fig. 9, E and G, were inadvertently changed to nM. These have been corrected to the original unit, μM, and Fig. 9 has been updated.

## References

[1] World Health Organization, WHO World Malaria Report 2022. https://who.int/teams/global-malaria-programme/reports/world-malaria-report-2022, (2022).

[2] C. V. Plowe, Malaria chemoprevention and drug resistance: A review of the literature and policy implications. Malar. J. 21, 104 (2022). 35331231 10.1186/s12936-022-04115-8 PMC8943514

[3] B. Hanboonkunupakarn, N. J. White, Advances and roadblocks in the treatment of malaria. Br. J. Clin. Pharmacol. 88, 374–382 (2022). 32656850 10.1111/bcp.14474 PMC9437935

[4] A. M. Dondorp, F. Nosten, P. Yi, D. Das, A. P. Phyo, J. Tarning, K. M. Lwin, F. Ariey, W. Hanpithakpong, S. J. Lee, P. Ringwald, K. Silamut, M. Imwong, K. Chotivanich, P. Lim, T. Herdman, S. S. An, S. Yeung, P. Singhasivanon, N. P. Day, N. Lindegardh, D. Socheat, N. J. White, Artemisinin resistance in *Plasmodium falciparum* malaria. N. Engl. J. Med. 361, 455–467 (2009). 19641202 10.1056/NEJMoa0808859 PMC3495232

[5] M. Imwong, M. Dhorda, K. M. Tun, A. M. Thu, A. P. Phyo, S. Proux, K. Suwannasin, C. Kunasol, S. Srisutham, J. Duanguppama, R. Vongpromek, C. Promnarate, A. Saejeng, N. Khantikul, R. Sugaram, S. Thanapongpichat, N. Sawangjaroen, K. Sutawong, K. T. Han, Y. Htut, K. Linn, A. A. Win, T. M. Hlaing, R. W. van der Pluijm, M. Mayxay, T. Pongvongsa, K. Phommasone, R. Tripura, T. J. Peto, L. von Seidlein, C. Nguon, D. Lek, X. H. S. Chan, H. Rekol, R. Leang, C. Huch, D. P. Kwiatkowski, O. Miotto, E. A. Ashley, M. P. Kyaw, S. Pukrittayakamee, N. P. J. Day, A. M. Dondorp, F. M. Smithuis, F. H. Nosten, N. J. White, Molecular epidemiology of resistance to antimalarial drugs in the Greater Mekong subregion: An observational study. Lancet Infect. Dis. 20, 1470–1480 (2020). 32679084 10.1016/S1473-3099(20)30228-0 PMC7689289

[6] M. Dhorda, C. Amaratunga, A. M. Dondorp, Artemisinin and multidrug-resistant *Plasmodium falciparum* - a threat for malaria control and elimination. Curr. Opin. Infect. Dis. 34, 432–439 (2021). 34267045 10.1097/QCO.0000000000000766 PMC8452334

[7] D. L. Saunders, P. Vanachayangkul, C. Lon; U.S. Army Military Malaria Research Program; National Center for Parasitology, Entomology, and Malaria Control (CNM); Royal Cambodian Armed Forces, Dihydroartemisinin-piperaquine failure in Cambodia. N. Engl. J. Med. 371, 484–485 (2014). 25075853 10.1056/NEJMc1403007

[8] R. W. van der Pluijm, M. Imwong, N. H. Chau, N. T. Hoa, N. T. Thuy-Nhien, N. V. Thanh, P. Jittamala, B. Hanboonkunupakarn, K. Chutasmit, C. Saelow, R. Runjarern, W. Kaewmok, R. Tripura, T. J. Peto, S. Yok, S. Suon, S. Sreng, S. Mao, S. Oun, S. Yen, C. Amaratunga, D. Lek, R. Huy, M. Dhorda, K. Chotivanich, E. A. Ashley, M. Mukaka, N. Waithira, P. Y. Cheah, R. J. Maude, R. Amato, R. D. Pearson, S. Goncalves, C. G. Jacob, W. L. Hamilton, R. M. Fairhurst, J. Tarning, M. Winterberg, D. P. Kwiatkowski, S. Pukrittayakamee, T. T. Hien, N. P. Day, O. Miotto, N. J. White, A. M. Dondorp, Determinants of dihydroartemisinin-piperaquine treatment failure in *Plasmodium falciparum* malaria in Cambodia, Thailand, and Vietnam: A prospective clinical, pharmacological, and genetic study. Lancet Infect. Dis. 19, 952–961 (2019). 31345710 10.1016/S1473-3099(19)30391-3 PMC6715822

[9] F. Ariey, B. Witkowski, C. Amaratunga, J. Beghain, A. C. Langlois, N. Khim, S. Kim, V. Duru, C. Bouchier, L. Ma, P. Lim, R. Leang, S. Duong, S. Sreng, S. Suon, C. M. Chuor, D. M. Bout, S. Menard, W. O. Rogers, B. Genton, T. Fandeur, O. Miotto, P. Ringwald, J. Le Bras, A. Berry, J. C. Barale, R. M. Fairhurst, F. Benoit-Vical, O. Mercereau-Puijalon, D. Menard, A molecular marker of artemisinin-resistant *Plasmodium falciparum* malaria. Nature 505, 50–55 (2014). 24352242 10.1038/nature12876 PMC5007947

[10] R. Amato, R. D. Pearson, J. Almagro-Garcia, C. Amaratunga, P. Lim, S. Suon, S. Sreng, E. Drury, J. Stalker, O. Miotto, R. M. Fairhurst, D. P. Kwiatkowski, Origins of the current outbreak of multidrug-resistant malaria in southeast Asia: A retrospective genetic study. Lancet Infect. Dis. 18, 337–345 (2018). 29398391 10.1016/S1473-3099(18)30068-9 PMC5835763

[11] F. A. Siddiqui, R. Boonhok, M. Cabrera, H. G. N. Mbenda, M. Wang, H. Min, X. Liang, J. Qin, X. Zhu, J. Miao, Y. Cao, L. Cui, Role of *Plasmodium falciparum* Kelch 13 protein mutations in *P. falciparum* populations from northeastern Myanmar in mediating artemisinin resistance. mBio 11, e01134-19 (2020). 32098812 10.1128/mBio.01134-19 PMC7042691

[12] B. H. Stokes, S. K. Dhingra, K. Rubiano, S. Mok, J. Straimer, N. F. Gnadig, I. Deni, K. A. Schindler, J. R. Bath, K. E. Ward, J. Striepen, T. Yeo, L. S. Ross, E. Legrand, F. Ariey, C. H. Cunningham, I. M. Souleymane, A. Gansane, R. Nzoumbou-Boko, C. Ndayikunda, A. M. Kabanywanyi, A. Uwimana, S. J. Smith, O. Kolley, M. Ndounga, M. Warsame, R. Leang, F. Nosten, T. J. Anderson, P. J. Rosenthal, D. Menard, D. A. Fidock, *Plasmodium falciparum* K13 mutations in Africa and Asia impact artemisinin resistance and parasite fitness. eLife 10, e66277 (2021). 34279219 10.7554/eLife.66277 PMC8321553

[13] T. Yang, L. M. Yeoh, M. V. Tutor, M. W. Dixon, P. J. McMillan, S. C. Xie, J. L. Bridgford, D. L. Gillett, M. F. Duffy, S. A. Ralph, M. J. McConville, L. Tilley, S. A. Cobbold, Decreased K13 abundance reduces hemoglobin catabolism and proteotoxic stress, underpinning artemisinin resistance. Cell Rep. 29, 2917–2928.e5 (2019). 31775055 10.1016/j.celrep.2019.10.095

[14] J. Birnbaum, S. Scharf, S. Schmidt, E. Jonscher, W. A. M. Hoeijmakers, S. Flemming, C. G. Toenhake, M. Schmitt, R. Sabitzki, B. Bergmann, U. Frohlke, P. Mesen-Ramirez, A. Blancke Soares, H. Herrmann, R. Bartfai, T. Spielmann, A Kelch13-defined endocytosis pathway mediates artemisinin resistance in malaria parasites. Science 367, 51–59 (2020). 31896710 10.1126/science.aax4735

[15] S. Mok, B. H. Stokes, N. F. Gnadig, L. S. Ross, T. Yeo, C. Amaratunga, E. Allman, L. Solyakov, A. R. Bottrill, J. Tripathi, R. M. Fairhurst, M. Llinas, Z. Bozdech, A. B. Tobin, D. A. Fidock, Artemisinin-resistant K13 mutations rewire *Plasmodium falciparum's* intra-erythrocytic metabolic program to enhance survival. Nat. Commun. 12, 530 (2021). 33483501 10.1038/s41467-020-20805-w PMC7822823

[16] F. A. Siddiqui, X. Liang, L. Cui, *Plasmodium falciparum* resistance to ACTs: Emergence, mechanisms, and outlook. Int. J. Parasitol. Drugs Drug Resist. 16, 102–118 (2021). 34090067 10.1016/j.ijpddr.2021.05.007 PMC8188179

[17] C. O. Egwu, P. Perio, J. M. Augereau, I. Tsamesidis, F. Benoit-Vical, K. Reybier, Resistance to artemisinin in falciparum malaria parasites: A redox-mediated phenomenon. Free Radic. Biol. Med. 179, 317–327 (2022). 34416340 10.1016/j.freeradbiomed.2021.08.016

[18] K. E. Ward, D. A. Fidock, J. L. Bridgford, *Plasmodium falciparum* resistance to artemisinin-based combination therapies. Curr. Opin. Microbiol. 69, 102193 (2022). 36007459 10.1016/j.mib.2022.102193 PMC9847095

[19] A. Uwimana, N. Umulisa, M. Venkatesan, S. S. Svigel, Z. Zhou, T. Munyaneza, R. M. Habimana, A. Rucogoza, L. F. Moriarty, R. Sandford, E. Piercefield, I. Goldman, B. Ezema, E. Talundzic, M. A. Pacheco, A. A. Escalante, D. Ngamije, J. N. Mangala, M. Kabera, K. Munguti, M. Murindahabi, W. Brieger, C. Musanabaganwa, L. Mutesa, V. Udhayakumar, A. Mbituyumuremyi, E. S. Halsey, N. W. Lucchi, Association of *Plasmodium falciparum* kelch13 R561H genotypes with delayed parasite clearance in Rwanda: An open-label, single-arm, multicentre, therapeutic efficacy study. Lancet Infect. Dis. 21, 1120–1128 (2021). 33864801 10.1016/S1473-3099(21)00142-0 PMC10202849

[20] A. Uwimana, E. Legrand, B. H. Stokes, J. M. Ndikumana, M. Warsame, N. Umulisa, D. Ngamije, T. Munyaneza, J. B. Mazarati, K. Munguti, P. Campagne, A. Criscuolo, F. Ariey, M. Murindahabi, P. Ringwald, D. A. Fidock, A. Mbituyumuremyi, D. Menard, Emergence and clonal expansion of *in vitro* artemisinin-resistant *Plasmodium falciparum* kelch13 R561H mutant parasites in Rwanda. Nat. Med. 26, 1602–1608 (2020). 32747827 10.1038/s41591-020-1005-2 PMC7541349

[21] B. Balikagala, N. Fukuda, M. Ikeda, O. T. Katuro, S. I. Tachibana, M. Yamauchi, W. Opio, S. Emoto, D. A. Anywar, E. Kimura, N. M. Q. Palacpac, E. I. Odongo-Aginya, M. Ogwang, T. Horii, T. Mita, Evidence of artemisinin-resistant malaria in Africa. N. Engl. J. Med. 385, 1163–1171 (2021). 34551228 10.1056/NEJMoa2101746

[22] B. H. Stokes, K. E. Ward, D. A. Fidock, Evidence of artemisinin-resistant malaria in Africa. N. Engl. J. Med. 386, 1385–1386 (2022). 35388682 10.1056/NEJMc2117480 PMC9888016

[23] B. Witkowski, V. Duru, N. Khim, L. S. Ross, B. Saintpierre, J. Beghain, S. Chy, S. Kim, S. Ke, N. Kloeung, R. Eam, C. Khean, M. Ken, K. Loch, A. Bouillon, A. Domergue, L. Ma, C. Bouchier, R. Leang, R. Huy, G. Nuel, J. C. Barale, E. Legrand, P. Ringwald, D. A. Fidock, O. Mercereau-Puijalon, F. Ariey, D. Menard, A surrogate marker of piperaquine-resistant *Plasmodium falciparum* malaria: A phenotype-genotype association study. Lancet Infect. Dis. 17, 174–183 (2017). 27818097 10.1016/S1473-3099(16)30415-7 PMC5266792

[24] R. Amato, P. Lim, O. Miotto, C. Amaratunga, D. Dek, R. D. Pearson, J. Almagro-Garcia, A. T. Neal, S. Sreng, S. Suon, E. Drury, D. Jyothi, J. Stalker, D. P. Kwiatkowski, R. M. Fairhurst, Genetic markers associated with dihydroartemisinin-piperaquine failure in *Plasmodium falciparum* malaria in Cambodia: A genotype-phenotype association study. Lancet Infect. Dis. 17, 164–173 (2017). 27818095 10.1016/S1473-3099(16)30409-1 PMC5564489

[25] V. Duru, N. Khim, R. Leang, S. Kim, A. Domergue, N. Kloeung, S. Ke, S. Chy, R. Eam, C. Khean, K. Loch, M. Ken, D. Lek, J. Beghain, F. Ariey, P. J. Guerin, R. Huy, O. Mercereau-Puijalon, B. Witkowski, D. Menard, *Plasmodium falciparum* dihydroartemisinin-piperaquine failures in Cambodia are associated with mutant K13 parasites presenting high survival rates in novel piperaquine *in vitro* assays: Retrospective and prospective investigations. BMC Med. 13, 305 (2015). 26695060 10.1186/s12916-015-0539-5 PMC4688949

[26] S. Agrawal, K. A. Moser, L. Morton, M. P. Cummings, A. Parihar, A. Dwivedi, A. C. Shetty, E. F. Drabek, C. G. Jacob, P. P. Henrich, C. M. Parobek, K. Jongsakul, R. Huy, M. D. Spring, C. A. Lanteri, S. Chaorattanakawee, C. Lon, M. M. Fukuda, D. L. Saunders, D. A. Fidock, J. T. Lin, J. J. Juliano, C. V. Plowe, J. C. Silva, S. Takala-Harrison, Association of a novel mutation in the *Plasmodium falciparum* chloroquine resistance transporter with decreased piperaquine sensitivity. J Infect Dis 216, 468–476 (2017). 28931241 10.1093/infdis/jix334 PMC5853219

[27] L. S. Ross, S. K. Dhingra, S. Mok, T. Yeo, K. J. Wicht, K. Kumpornsin, S. Takala-Harrison, B. Witkowski, R. M. Fairhurst, F. Ariey, D. Menard, D. A. Fidock, Emerging Southeast Asian PfCRT mutations confer *Plasmodium falciparum* resistance to the first-line antimalarial piperaquine. Nat. Commun. 9, 3314 (2018). 30115924 10.1038/s41467-018-05652-0 PMC6095916

[28] W. L. Hamilton, R. Amato, R. W. van der Pluijm, C. G. Jacob, H. H. Quang, N. T. Thuy-Nhien, T. T. Hien, B. Hongvanthong, K. Chindavongsa, M. Mayxay, R. Huy, R. Leang, C. Huch, L. Dysoley, C. Amaratunga, S. Suon, R. M. Fairhurst, R. Tripura, T. J. Peto, Y. Sovann, P. Jittamala, B. Hanboonkunupakarn, S. Pukrittayakamee, N. H. Chau, M. Imwong, M. Dhorda, R. Vongpromek, X. H. S. Chan, R. J. Maude, R. D. Pearson, T. Nguyen, K. Rockett, E. Drury, S. Goncalves, N. J. White, N. P. Day, D. P. Kwiatkowski, A. M. Dondorp, O. Miotto, Evolution and expansion of multidrug-resistant malaria in southeast Asia: A genomic epidemiology study. Lancet Infect. Dis. 19, 943–951 (2019). 31345709 10.1016/S1473-3099(19)30392-5 PMC6715858

[29] J. M. Sa, O. Twu, K. Hayton, S. Reyes, M. P. Fay, P. Ringwald, T. E. Wellems, Geographic patterns of *Plasmodium falciparum* drug resistance distinguished by differential responses to amodiaquine and chloroquine. Proc. Natl. Acad. Sci. U.S.A. 106, 18883–18889 (2009). 19884511 10.1073/pnas.0911317106 PMC2771746

[30] K. Beshir, C. J. Sutherland, I. Merinopoulos, N. Durrani, T. Leslie, M. Rowland, R. L. Hallett, Amodiaquine resistance in *Plasmodium falciparum* malaria in Afghanistan is associated with the *pfcrt* SVMNT allele at codons 72 to 76. Antimicrob. Agents Chemother. 54, 3714–3716 (2010). 20547800 10.1128/AAC.00358-10 PMC2934991

[31] M. Venkatesan, N. B. Gadalla, K. Stepniewska, P. Dahal, C. Nsanzabana, C. Moriera, R. N. Price, A. Martensson, P. J. Rosenthal, G. Dorsey, C. J. Sutherland, P. Guerin, T. M. E. Davis, D. Menard, I. Adam, G. Ademowo, C. Arze, F. N. Baliraine, N. Berens-Riha, A. Bjorkman, S. Borrmann, F. Checchi, M. Desai, M. Dhorda, A. A. Djimde, B. B. El-Sayed, T. Eshetu, F. Eyase, C. Falade, J. F. Faucher, G. Froberg, A. Grivoyannis, S. Hamour, S. Houze, J. Johnson, E. Kamugisha, S. Kariuki, J. R. Kiechel, F. Kironde, P. E. Kofoed, J. LeBras, M. Malmberg, L. Mwai, B. Ngasala, F. Nosten, S. L. Nsobya, A. Nzila, M. Oguike, S. D. Otienoburu, B. Ogutu, J. B. Ouedraogo, P. Piola, L. Rombo, B. Schramm, A. F. Some, J. Thwing, J. Ursing, R. P. M. Wong, A. Zeynudin, I. Zongo, C. V. Plowe, C. H. Sibley; Asaq Molecular Marker Study Group, Polymorphisms in *Plasmodium falciparum* chloroquine resistance transporter and multidrug resistance 1 genes: Parasite risk factors that affect treatment outcomes for *P. falciparum* malaria after artemether-lumefantrine and artesunate-amodiaquine. Am. J. Trop. Med. Hyg. 91, 833–843 (2014). 25048375 10.4269/ajtmh.14-0031 PMC4183414

[32] J. Kim, Y. Z. Tan, K. J. Wicht, S. K. Erramilli, S. K. Dhingra, J. Okombo, J. Vendome, L. M. Hagenah, S. I. Giacometti, A. L. Warren, K. Nosol, P. D. Roepe, C. S. Potter, B. Carragher, A. A. Kossiakoff, M. Quick, D. A. Fidock, F. Mancia, Structure and drug resistance of the *Plasmodium falciparum* transporter PfCRT. Nature 576, 315–320 (2019). 31776516 10.1038/s41586-019-1795-x PMC6911266

[33] B. Riegel, P. D. Roepe, Altered drug transport by *Plasmodium falciparum* chloroquine resistance transporter isoforms harboring mutations associated with piperaquine resistance. Biochemistry 59, 2484–2493 (2020). 32589406 10.1021/acs.biochem.0c00247

[34] S. K. Dhingra, D. Redhi, J. M. Combrinck, T. Yeo, J. Okombo, P. P. Henrich, A. N. Cowell, P. Gupta, M. L. Stegman, J. M. Hoke, R. A. Cooper, E. Winzeler, S. Mok, T. J. Egan, D. A. Fidock, A variant PfCRT isoform can contribute to *Plasmodium falciparum* resistance to the first-line partner drug piperaquine. mBio 8, e00303–e00317 (2017). 28487425 10.1128/mBio.00303-17 PMC5424201

[35] K. J. Wicht, S. Mok, D. A. Fidock, Molecular mechanisms of drug resistance in *Plasmodium falciparum* malaria. Annu. Rev. Microbiol. 74, 431–454 (2020). 32905757 10.1146/annurev-micro-020518-115546 PMC8130186

[36] G. M. Gomez, G. D'Arrigo, C. P. Sanchez, F. Berger, R. C. Wade, M. Lanzer, PfCRT mutations conferring piperaquine resistance in *falciparum* malaria shape the kinetics of quinoline drug binding and transport. PLOS Pathog. 19, e1011436 (2023). 37285379 10.1371/journal.ppat.1011436 PMC10281575

[37] S. Bopp, P. Magistrado, W. Wong, S. F. Schaffner, A. Mukherjee, P. Lim, M. Dhorda, C. Amaratunga, C. J. Woodrow, E. A. Ashley, N. J. White, A. M. Dondorp, R. M. Fairhurst, F. Ariey, D. Menard, D. F. Wirth, S. K. Volkman, Plasmepsin II-III copy number accounts for bimodal piperaquine resistance among Cambodian *Plasmodium falciparum*. Nat. Commun. 9, 1769 (2018). 29720620 10.1038/s41467-018-04104-z PMC5931971

[38] X. Z. Su, K. D. Lane, L. Xia, J. M. Sa, T. E. Wellems, *Plasmodium* genomics and genetics: New insights into malaria pathogenesis, drug resistance, epidemiology, and evolution. Clin. Microbiol. Rev. 32, e00019–e00019 (2019). 31366610 10.1128/CMR.00019-19 PMC6750138

[39] A. M. Vaughan, R. S. Pinapati, I. H. Cheeseman, N. Camargo, M. Fishbaugher, L. A. Checkley, S. Nair, C. A. Hutyra, F. H. Nosten, T. J. Anderson, M. T. Ferdig, S. H. Kappe, *Plasmodium falciparum* genetic crosses in a humanized mouse model. Nat. Methods 12, 631–633 (2015). 26030447 10.1038/nmeth.3432 PMC4547688

[40] K. A. Button-Simons, S. Kumar, N. Carmago, M. T. Haile, C. Jett, L. A. Checkley, S. Y. Kennedy, R. S. Pinapati, D. A. Shoue, M. McDew-White, X. Li, F. H. Nosten, S. H. Kappe, T. J. C. Anderson, J. Romero-Severson, M. T. Ferdig, S. J. Emrich, A. M. Vaughan, I. H. Cheeseman, The power and promise of genetic mapping from *Plasmodium falciparum* crosses utilizing human liver-chimeric mice. Commun. Biol. 4, 734 (2021). 34127785 10.1038/s42003-021-02210-1 PMC8203791

[41] K. V. Brenneman, X. Li, S. Kumar, E. Delgado, L. A. Checkley, D. A. Shoue, A. Reyes, B. A. Abatiyow, M. T. Haile, R. Tripura, T. Peto, D. Lek, K. A. Button-Simons, S. H. I. Kappe, M. Dhorda, F. Nosten, S. C. Nkhoma, I. H. Cheeseman, A. M. Vaughan, M. T. Ferdig, T. J. C. Anderson, Optimizing bulk segregant analysis of drug resistance using *Plasmodium falciparum* genetic crosses conducted in humanized mice. iScience 25, 104095 (2022). 35372813 10.1016/j.isci.2022.104095 PMC8971943

[42] X. Li, S. Kumar, K. V. Brenneman, T. J. C. Anderson, Bulk segregant linkage mapping for rodent and human malaria parasites. Parasitol. Int. 91, 102653 (2022). 36007706 10.1016/j.parint.2022.102653 PMC11972598

[43] A. Amambua-Ngwa, K. A. Button-Simons, X. Li, S. Kumar, K. V. Brenneman, M. Ferrari, L. A. Checkley, M. T. Haile, D. A. Shoue, M. McDew-White, S. M. Tindall, A. Reyes, E. Delgado, H. Dalhoff, J. K. Larbalestier, R. Amato, R. D. Pearson, A. B. Taylor, F. H. Nosten, U. D'Alessandro, D. Kwiatkowski, I. H. Cheeseman, S. H. I. Kappe, S. V. Avery, D. J. Conway, A. M. Vaughan, M. T. Ferdig, T. J. C. Anderson, Chloroquine resistance evolution in *Plasmodium falciparum* is mediated by the putative amino acid transporter AAT1. Nat. Microbiol. 8, 1213–1226 (2023). 37169919 10.1038/s41564-023-01377-z PMC10322710

[44] X. Su, M. T. Ferdig, Y. Huang, C. Q. Huynh, A. Liu, J. You, J. C. Wootton, T. E. Wellems, A genetic map and recombination parameters of the human malaria parasite *Plasmodium falciparum*. Science 286, 1351–1353 (1999). 10558988 10.1126/science.286.5443.1351

[45] A. Miles, Z. Iqbal, P. Vauterin, R. Pearson, S. Campino, M. Theron, K. Gould, D. Mead, E. Drury, J. O'Brien, V. Ruano Rubio, B. MacInnis, J. Mwangi, U. Samarakoon, L. Ranford-Cartwright, M. Ferdig, K. Hayton, X. Z. Su, T. Wellems, J. Rayner, G. McVean, D. Kwiatkowski, Indels, structural variation, and recombination drive genomic diversity in *Plasmodium falciparum*. Genome Res. 26, 1288–1299 (2016). 27531718 10.1101/gr.203711.115 PMC5052046

[46] M. M. MalariaGen, M. H. Abdel Hamid, D. O. Abdelraheem, A. Acheampong, M. Ahouidi, J. Ali, A. Almagro-Garcia, C. Amambua-Ngwa, L. Amaratunga, B. Amenga-Etego, T. Andagalu, V. Anderson, I. Andrianaranjaka, E. Aniebo, F. Aninagyei, P. O. Ansah, T. Ansah, P. Apinjoh, E. Arnaldo, S. Ashley, G. A. Auburn, H. Awandare, V. Ba, A. Baraka, P. Barry, G. I. Bejon, M. F. Bertin, S. Boni, T. Borrmann, M. Bousema, O. Bouyou-Akotet, P. C. Branch, H. Bull, K. Cheah, T. Chindavongsa, K. Chookajorn, A. Chotivanich, D. J. Claessens, V. Conway, E. Corredor, A. Courtier, U. Craig, S. D'Alessandro, N. Dama, B. Day, M. Denis, M. Dhorda, A. Diakite, C. Djimde, A. Dolecek, S. Dondorp, C. Doumbia, E. Drakeley, P. Drury, D. F. Duffy, T. G. Echeverry, S. M. M. Egwang, B. Enosse, R. M. Erko, A. Fairhurst, C. A. Faiz, M. Fanello, M. Fleharty, M. Forbes, D. Fukuda, A. Gamboa, L. Ghansah, S. Golassa, G. L. A. Goncalves, S. A. Harrison, J. A. Healy, A. Hendry, T. T. Hernandez-Koutoucheva, C. A. Hien, F. Hill, A. Hombhanje, Y. Hott, M. Htut, M. Hussein, D. Imwong, S. A. Ishengoma, C. G. Jackson, J. Jacob, K. J. Jeans, C. Johnson, E. Kamaliddin, J. Kamau, T. Keatley, D. S. Kochakarn, A. Konate, A. Konate, D. P. Kone, M. P. Kwiatkowski, D. Kyaw, M. Kyle, S. K. Lawniczak, M. Lee, P. Lemnge, C. Lim, K. M. Lon, C. I. Loua, J. Mandara, K. Marfurt, R. J. Marsh, M. Maude, O. Mayxay, O. Maiga-Ascofare, T. Miotto, V. Mita, A. O. Mobegi, O. A. Mohamed, J. Mokuolu, C. M. Montgomery, I. Morang'a, K. Mueller, P. N. Murie, T. N. Newton, T. Duc, T. N. Nguyen, T. N. Nguyen, H. Thi Kim, H. Nguyen Van, F. Noedl, R. Nosten, V. N. Noviyanti, A. Ntui, L. I. Nzila, H. Ochola-Oyier, A. Ocholla, I. Oduro, M. A. Omedo, J. B. Onyamboko, K. Ouedraogo, W. A. Oyebola, R. Oyibo, N. Pearson, A. P. Peshu, C. V. Phyo, R. N. Plowe, S. Price, H. H. Pukrittayakamee, M. Quang, J. C. Randrianarivelojosia, P. Rayner, A. Ringwald, E. Rosanas-Urgell, V. Rovira-Vallbona, L. Ruano-Rubio, D. Ruiz, A. Saunders, P. Shayo, V. J. Siba, M. S. Simpson, C. Sissoko, X. Smith, Z. Su, C. Sutherland, S. Takala-Harrison, A. Talman, L. Tavul, N. V. Thanh, V. Thathy, A. M. Thu, M. Toure, A. Tshefu, F. Verra, J. Vinetz, T. E. Wellems, J. Wendler, N. J. White, G. Whitton, W. Yavo, R. W. van der Pluijm, Pf7: An open dataset of *Plasmodium falciparum* genome variation in 20,000 worldwide samples. Wellcome Open Res. 8, 22 (2023). 36864926 10.12688/wellcomeopenres.18681.1 PMC9971654

[47] Y. Yuthavong, B. Tarnchompoo, T. Vilaivan, P. Chitnumsub, S. Kamchonwongpaisan, S. A. Charman, D. N. McLennan, K. L. White, L. Vivas, E. Bongard, C. Thongphanchang, S. Taweechai, J. Vanichtanankul, R. Rattanajak, U. Arwon, P. Fantauzzi, J. Yuvaniyama, W. N. Charman, D. Matthews, Malarial dihydrofolate reductase as a paradigm for drug development against a resistance-compromised target. Proc. Natl. Acad. Sci. U.S.A. 109, 16823–16828 (2012). 23035243 10.1073/pnas.1204556109 PMC3479511

[48] A. N. Cowell, E. S. Istvan, A. K. Lukens, M. G. Gomez-Lorenzo, M. Vanaerschot, T. Sakata-Kato, E. L. Flannery, P. Magistrado, E. Owen, M. Abraham, G. LaMonte, H. J. Painter, R. M. Williams, V. Franco, M. Linares, I. Arriaga, S. Bopp, V. C. Corey, N. F. Gnadig, O. Coburn-Flynn, C. Reimer, P. Gupta, J. M. Murithi, P. A. Moura, O. Fuchs, E. Sasaki, S. W. Kim, C. H. Teng, L. T. Wang, A. Akidil, S. Adjalley, P. A. Willis, D. Siegel, O. Tanaseichuk, Y. Zhong, Y. Zhou, M. Llinas, S. Ottilie, F. J. Gamo, M. C. S. Lee, D. E. Goldberg, D. A. Fidock, D. F. Wirth, E. A. Winzeler, Mapping the malaria parasite druggable genome by using *in vitro* evolution and chemogenomics. Science 359, 191–199 (2018). 29326268 10.1126/science.aan4472 PMC5925756

[49] J. M. Murithi, I. Deni, C. F. A. Pasaje, J. Okombo, J. L. Bridgford, N. F. Gnadig, R. L. Edwards, T. Yeo, S. Mok, A. Y. Burkhard, O. Coburn-Flynn, E. S. Istvan, T. Sakata-Kato, M. G. Gomez-Lorenzo, A. N. Cowell, K. J. Wicht, C. Le Manach, G. F. Kalantarov, S. Dey, M. Duffey, B. Laleu, A. K. Lukens, S. Ottilie, M. Vanaerschot, I. N. Trakht, F. J. Gamo, D. F. Wirth, D. E. Goldberg, A. R. Odom John, K. Chibale, E. A. Winzeler, J. C. Niles, D. A. Fidock, The *Plasmodium falciparum* ABC transporter ABCI3 confers parasite strain-dependent pleiotropic antimalarial drug resistance. Chem. Biol. 29, 824–839.e6 (2022). 10.1016/j.chembiol.2021.06.006 PMC8727639 34233174

[50] MalariaGEN *Plasmodium falciparum* Community Project, Genomic epidemiology of artemisinin resistant malaria. eLife 5, e08714 (2016). 26943619 10.7554/eLife.08714 PMC4786412

[51] S. Bellanca, R. L. Summers, M. Meyrath, A. Dave, M. N. Nash, M. Dittmer, C. P. Sanchez, W. D. Stein, R. E. Martin, M. Lanzer, Multiple drugs compete for transport via the *Plasmodium falciparum* chloroquine resistance transporter at distinct but interdependent sites. J. Biol. Chem. 289, 36336–36351 (2014). 25378409 10.1074/jbc.M114.614206 PMC4276893

[52] D. A. Fidock, P. J. Rosenthal, Artemisinin resistance in Africa: How urgent is the threat? Med 2, 1287–1288 (2021). 35005674 10.1016/j.medj.2021.11.005 PMC8729812

[53] N. J. White, Severe malaria. Malar. J. 21, 284 (2022). 36203155 10.1186/s12936-022-04301-8 PMC9536054

[54] Z. Wang, Y. Wang, M. Cabrera, Y. Zhang, B. Gupta, Y. Wu, K. Kemirembe, Y. Hu, X. Liang, A. Brashear, S. Shrestha, X. Li, J. Miao, X. Sun, Z. Yang, L. Cui, Artemisinin resistance at the China-Myanmar border and association with mutations in the K13 propeller gene. Antimicrob. Agents Chemother. 59, 6952–6959 (2015). 26324266 10.1128/AAC.01255-15 PMC4604380

[55] Y. Zhao, Z. Liu, M. T. Soe, L. Wang, T. N. Soe, H. Wei, A. Than, P. L. Aung, Y. Li, X. Zhang, Y. Hu, H. Wei, Y. Zhang, J. Burgess, F. A. Siddiqui, L. Menezes, Q. Wang, M. P. Kyaw, Y. Cao, L. Cui, Genetic variations associated with drug resistance markers in asymptomatic *Plasmodium falciparum* infections in Myanmar. Genes 10, 692 (2019). 31505774 10.3390/genes10090692 PMC6770986

[56] S. Dahlstrom, P. E. Ferreira, M. I. Veiga, N. Sedighi, L. Wiklund, A. Martensson, A. Farnert, C. Sisowath, L. Osorio, H. Darban, B. Andersson, A. Kaneko, G. Conseil, A. Bjorkman, J. P. Gil, *Plasmodium* falciparum multidrug resistance protein 1 and artemisinin-based combination therapy in Africa. J. Infect. Dis. 200, 1456–1464 (2009). 19807279 10.1086/606009

[57] D. K. Raj, J. Mu, H. Jiang, J. Kabat, S. Singh, M. Sullivan, M. P. Fay, T. F. McCutchan, X. Z. Su, Disruption of a *Plasmodium falciparum* multidrug resistance-associated protein (PfMRP) alters its fitness and transport of antimalarial drugs and glutathione. J. Biol. Chem. 284, 7687–7696 (2009). 19117944 10.1074/jbc.M806944200 PMC2658063

[58] O. Miotto, R. Amato, E. A. Ashley, B. MacInnis, J. Almagro-Garcia, C. Amaratunga, P. Lim, D. Mead, S. O. Oyola, M. Dhorda, M. Imwong, C. Woodrow, M. Manske, J. Stalker, E. Drury, S. Campino, L. Amenga-Etego, T. N. Thanh, H. T. Tran, P. Ringwald, D. Bethell, F. Nosten, A. P. Phyo, S. Pukrittayakamee, K. Chotivanich, C. M. Chuor, C. Nguon, S. Suon, S. Sreng, P. N. Newton, M. Mayxay, M. Khanthavong, B. Hongvanthong, Y. Htut, K. T. Han, M. P. Kyaw, M. A. Faiz, C. I. Fanello, M. Onyamboko, O. A. Mokuolu, C. G. Jacob, S. Takala-Harrison, C. V. Plowe, N. P. Day, A. M. Dondorp, C. C. Spencer, G. McVean, R. M. Fairhurst, N. J. White, D. P. Kwiatkowski, Genetic architecture of artemisinin-resistant *Plasmodium falciparum*. Nat. Genet. 47, 226–234 (2015). 25599401 10.1038/ng.3189 PMC4545236

[59] T. Kampoun, S. Srichairatanakool, P. Prommana, P. J. Shaw, J. L. Green, E. Knuepfer, A. A. Holder, C. Uthaipibull, Apicoplast ribosomal protein S10-V127M enhances artemisinin resistance of a Kelch13 transgenic *Plasmodium falciparum*. Malar. J. 21, 302 (2022). 36303209 10.1186/s12936-022-04330-3 PMC9615251

[60] X. Li, S. Kumar, M. McDew-White, M. Haile, I. H. Cheeseman, S. Emrich, K. Button-Simons, F. Nosten, S. H. I. Kappe, M. T. Ferdig, T. J. C. Anderson, A. M. Vaughan, Genetic mapping of fitness determinants across the malaria parasite *Plasmodium falciparum* life cycle. PLOS Genet. 15, e1008453 (2019). 31609965 10.1371/journal.pgen.1008453 PMC6821138

[61] R. M. Hoglund, L. Workman, M. D. Edstein, N. X. Thanh, N. N. Quang, I. Zongo, J. B. Ouedraogo, S. Borrmann, L. Mwai, C. Nsanzabana, R. N. Price, P. Dahal, N. C. Sambol, S. Parikh, F. Nosten, E. A. Ashley, A. P. Phyo, K. M. Lwin, R. McGready, N. P. Day, P. J. Guerin, N. J. White, K. I. Barnes, J. Tarning, Population pharmacokinetic properties of piperaquine in *falciparum* malaria: An individual participant data meta-Analysis. PLOS Med. 14, e1002212 (2017). 28072872 10.1371/journal.pmed.1002212 PMC5224788

[62] M. Imwong, K. Suwannasin, S. Srisutham, R. Vongpromek, C. Promnarate, A. Saejeng, A. P. Phyo, S. Proux, T. Pongvongsa, N. Chea, O. Miotto, R. Tripura, C. Nguyen Hoang, L. Dysoley, N. H. D. Trung, T. J. Peto, J. J. Callery, R. W. van der Pluijm, C. Amaratunga, M. Mukaka, L. von Seidlein, M. Mayxay, N. T. Thuy-Nhien, P. N. Newton, N. P. J. Day, E. A. Ashley, F. H. Nosten, F. M. Smithuis, M. Dhorda, N. J. White, A. M. Dondorp, Evolution of multidrug resistance in *Plasmodium falciparum*: A longitudinal study of genetic resistance markers in the Greater Mekong subregion. Antimicrob. Agents Chemother. 65, e0112121 (2021). 34516247 10.1128/AAC.01121-21 PMC8597770

[63] S. N. Richards, M. N. Nash, E. S. Baker, M. W. Webster, A. M. Lehane, S. H. Shafik, R. E. Martin, Molecular mechanisms for drug hypersensitivity induced by the malaria parasite's chloroquine resistance transporter. PLOS Pathog. 12, e1005725 (2016). 27441371 10.1371/journal.ppat.1005725 PMC4956231

[64] S. H. Shafik, S. N. Richards, B. Corry, R. E. Martin, Mechanistic basis for multidrug resistance and collateral drug sensitivity conferred to the malaria parasite by polymorphisms in PfMDR1 and PfCRT. PLOS Biol. 20, e3001616 (2022). 35507548 10.1371/journal.pbio.3001616 PMC9067703

[65] P. Rohrbach, C. P. Sanchez, K. Hayton, O. Friedrich, J. Patel, A. B. Sidhu, M. T. Ferdig, D. A. Fidock, M. Lanzer, Genetic linkage of *pfmdr1* with food vacuolar solute import in *Plasmodium falciparum*. EMBO J. 25, 3000–3011 (2006). 16794577 10.1038/sj.emboj.7601203 PMC1500988

[66] M. Chugh, V. Sundararaman, S. Kumar, V. S. Reddy, W. A. Siddiqui, K. D. Stuart, P. Malhotra, Protein complex directs hemoglobin-to-hemozoin formation in *Plasmodium falciparum*. Proc. Natl. Acad. Sci. U.S.A. 110, 5392–5397 (2013). 23471987 10.1073/pnas.1218412110 PMC3619337

[67] D. Loesbanluechai, N. Kotanan, C. de Cozar, T. Kochakarn, M. R. Ansbro, K. Chotivanich, N. J. White, P. Wilairat, M. C. S. Lee, F. J. Gamo, L. M. Sanz, T. Chookajorn, K. Kumpornsin, Overexpression of plasmepsin II and plasmepsin III does not directly cause reduction in *Plasmodium falciparum* sensitivity to artesunate, chloroquine and piperaquine. Int. J. Parasitol. Drugs Drug Resist. 9, 16–22 (2019). 30580023 10.1016/j.ijpddr.2018.11.004 PMC6304341

[68] S. K. Dhingra, J. L. Small-Saunders, D. Menard, D. A. Fidock, *Plasmodium falciparum* resistance to piperaquine driven by PfCRT. Lancet Infect. Dis. 19, 1168–1169 (2019). 31657776 10.1016/S1473-3099(19)30543-2 PMC6943240

[69] K. J. Wicht, J. L. Small-Saunders, L. M. Hagenah, S. Mok, D. A. Fidock, Mutant PfCRT can mediate piperaquine resistance in African *Plasmodium falciparum* with reduced fitness and increased susceptibility to other antimalarials. J. Infect. Dis. 226, 2021–2029 (2022). 36082431 10.1093/infdis/jiac365 PMC9704436

[70] S. H. Shafik, S. A. Cobbold, K. Barkat, S. N. Richards, N. S. Lancaster, M. Llinas, S. J. Hogg, R. L. Summers, M. J. McConville, R. E. Martin, The natural function of the malaria parasite's chloroquine resistance transporter. Nat. Commun. 11, 3922 (2020). 32764664 10.1038/s41467-020-17781-6 PMC7413254

[71] J. Okombo, S. Mok, T. Qahash, T. Yeo, J. Bath, L. M. Orchard, E. Owens, I. Koo, I. Albert, M. Llinas, D. A. Fidock, Piperaquine-resistant PfCRT mutations differentially impact drug transport, hemoglobin catabolism and parasite physiology in *Plasmodium falciparum* asexual blood stages. PLOS Pathog. 18, e1010926 (2022). 36306287 10.1371/journal.ppat.1010926 PMC9645663

[72] C. P. Sanchez, E. D. T. Manson, S. Moliner Cubel, L. Mandel, S. K. Weidt, M. P. Barrett, M. Lanzer, The knock-down of the chloroquine resistance transporter PfCRT is linked to oligopeptide handling in *Plasmodium falciparum*. Microbiol. Spectr. 10, e0110122 (2022). 35867395 10.1128/spectrum.01101-22 PMC9431119

[73] P. Tumwebaze, S. Tukwasibwe, A. Taylor, M. Conrad, E. Ruhamyankaka, V. Asua, A. Walakira, J. Nankabirwa, A. Yeka, S. G. Staedke, B. Greenhouse, S. L. Nsobya, M. R. Kamya, G. Dorsey, P. J. Rosenthal, Changing antimalarial drug resistance patterns identified by surveillance at three sites in Uganda. J Infect Dis 215, 631–635 (2017). 28039354 10.1093/infdis/jiw614 PMC5853976

[74] P. Chotsiri, N. J. White, J. Tarning, Pharmacokinetic considerations in seasonal malaria chemoprevention. Trends Parasitol. 38, 673–682 (2022). 35688778 10.1016/j.pt.2022.05.003

[75] R. W. van der Pluijm, R. Tripura, R. M. Hoglund, A. P. Phyo, D. Lek, A. U. Islam, A. R. Anvikar, P. Satpathi, S. Satpathi, P. K. Behera, A. Tripura, S. Baidya, M. Onyamboko, N. H. Chau, Y. Sovann, S. Suon, S. Sreng, S. Mao, S. Oun, S. Yen, C. Amaratunga, K. Chutasmit, C. Saelow, R. Runcharern, W. Kaewmok, N. T. Hoa, N. V. Thanh, B. Hanboonkunupakarn, J. J. Callery, A. K. Mohanty, J. Heaton, M. Thant, K. Gantait, T. Ghosh, R. Amato, R. D. Pearson, C. G. Jacob, S. Goncalves, M. Mukaka, N. Waithira, C. J. Woodrow, M. P. Grobusch, M. van Vugt, R. M. Fairhurst, P. Y. Cheah, T. J. Peto, L. von Seidlein, M. Dhorda, R. J. Maude, M. Winterberg, N. T. Thuy-Nhien, D. P. Kwiatkowski, M. Imwong, P. Jittamala, K. Lin, T. M. Hlaing, K. Chotivanich, R. Huy, C. Fanello, E. Ashley, M. Mayxay, P. N. Newton, T. T. Hien, N. Valecha, F. Smithuis, S. Pukrittayakamee, A. Faiz, O. Miotto, J. Tarning, N. P. J. Day, N. J. White, A. M. Dondorp; Tracking Resistance to Artemisinin Collaboration, Triple artemisinin-based combination therapies versus artemisinin-based combination therapies for uncomplicated *Plasmodium falciparum* malaria: A multicentre, open-label, randomised clinical trial. Lancet 395, 1345–1360 (2020). 32171078 10.1016/S0140-6736(20)30552-3 PMC8204272

[76] A. Amambua-Ngwa, L. Amenga-Etego, E. Kamau, R. Amato, A. Ghansah, L. Golassa, M. Randrianarivelojosia, D. Ishengoma, T. Apinjoh, O. Maiga-Ascofare, B. Andagalu, W. Yavo, M. Bouyou-Akotet, O. Kolapo, K. Mane, A. Worwui, D. Jeffries, V. Simpson, U. D'Alessandro, D. Kwiatkowski, A. A. Djimde, Major subpopulations of *Plasmodium falciparum* in sub-Saharan Africa. Science 365, 813–816 (2019). 31439796 10.1126/science.aav5427

[77] A. K. Tripathi, G. Mlambo, S. Kanatani, P. Sinnis, G. Dimopoulos, *Plasmodium falciparum* gametocyte culture and mosquito infection through artificial membrane feeding. J. Vis. Exp. 3, 10.3791/61426 (2020). 10.3791/61426 PMC8216103 32716382

[78] R. Xi, A. G. Hadjipanayis, L. J. Luquette, T. M. Kim, E. Lee, J. Zhang, M. D. Johnson, D. M. Muzny, D. A. Wheeler, R. A. Gibbs, R. Kucherlapati, P. J. Park, Copy number variation detection in whole-genome sequencing data using the Bayesian information criterion. Proc. Natl. Acad. Sci. U.S.A. 108, E1128–E1136 (2011). 22065754 10.1073/pnas.1110574108 PMC3219132

[79] M. Kanai, T. Yeo, V. Asua, P. J. Rosenthal, D. A. Fidock, S. Mok, Comparative analysis of *Plasmodium falciparum* genotyping via SNP detection, microsatellite profiling, and whole-genome sequencing. Antimicrob. Agents Chemother. 66, e0116321 (2022). 34694871 10.1128/AAC.01163-21 PMC8765236

[80] M. Imwong, K. Suwannasin, C. Kunasol, K. Sutawong, M. Mayxay, H. Rekol, F. M. Smithuis, T. M. Hlaing, K. M. Tun, R. W. van der Pluijm, R. Tripura, O. Miotto, D. Menard, M. Dhorda, N. P. J. Day, N. J. White, A. M. Dondorp, The spread of artemisinin-resistant *Plasmodium falciparum* in the Greater Mekong subregion: A molecular epidemiology observational study. Lancet Infect. Dis. 17, 491–497 (2017). 28161569 10.1016/S1473-3099(17)30048-8 PMC5406483

[81] B. Witkowski, C. Amaratunga, N. Khim, S. Sreng, P. Chim, S. Kim, P. Lim, S. Mao, C. Sopha, B. Sam, J. M. Anderson, S. Duong, C. M. Chuor, W. R. Taylor, S. Suon, O. Mercereau-Puijalon, R. M. Fairhurst, D. Menard, Novel phenotypic assays for the detection of artemisinin-resistant *Plasmodium falciparum* malaria in Cambodia: *In-vitro* and *ex-vivo* drug-response studies. Lancet Infect. Dis. 13, 1043–1049 (2013). 24035558 10.1016/S1473-3099(13)70252-4 PMC5015432

[82] C. Amaratunga, A. T. Neal, R. M. Fairhurst, Flow cytometry-based analysis of artemisinin-resistant *Plasmodium falciparum* in the ring-stage survival assay. Antimicrob. Agents Chemother. 58, 4938–4940 (2014). 24867976 10.1128/AAC.02902-14 PMC4136004

[83] K. W. Broman, H. Wu, S. Sen, G. A. Churchill, R/qtl: QTL mapping in experimental crosses. Bioinformatics 19, 889–890 (2003). 12724300 10.1093/bioinformatics/btg112

[84] J. W. Van Ooijen, JoinMap® 5, software for the calculation of genetic linkage maps in experimental populations of diploid species. Kyazma B.V., (Wageningen, Netherlands 2018).

[85] B. N. Mansfeld, R. Grumet, QTLseqr: An R package for bulk segregant analysis with next-generation sequencing. Plant Genome 11, 1–5 (2018). 10.3835/plantgenome2018.01.0006 PMC12810111 30025013

[86] K. W. Broman, D. M. Gatti, P. Simecek, N. A. Furlotte, P. Prins, S. Sen, B. S. Yandell, G. A. Churchill, R/qtl2: Software for mapping quantitative trait loci with high-dimensional data and multiparent populations. Genetics 211, 495–502 (2019). 30591514 10.1534/genetics.118.301595 PMC6366910

[87] S. J. Gabryszewski, C. Modchang, L. Musset, T. Chookajorn, D. A. Fidock, Combinatorial genetic modeling of *pfcrt*-mediated drug resistance evolution in *Plasmodium falciparum*. Mol. Biol. Evol. 33, 1554–1570 (2016). 26908582 10.1093/molbev/msw037 PMC4868112

